# Adsorption and Sensing Behavior of Pristine, P‐Doped, and Al‐Doped Boron Nitride Nanosheets Toward Toxic Hydrogen Fluoride Gas: Insights from Density Functional Theory Analysis

**DOI:** 10.1002/open.202500560

**Published:** 2026-04-20

**Authors:** Nihal Siddique, Rashek Dewan Daymond, Md. Hasan Shahria Fahim, Utso Jyoti Golder, Debashis Roy, Abdullah Al Roman, Mohammad Tanvir Ahmed

**Affiliations:** ^1^ Condensed Matter Physics (CMP) Lab, Department of Physics Jashore University of Science and Technology Jashore 7408 Bangladesh

**Keywords:** Two‐dimensional (2D) material, boron nitride, density functional theory (DFT), gas sensing, sensitivity

## Abstract

This study employs first‐principles density functional theory (DFT) to investigate the adsorption behavior of pristine, P‐doped, and Al‐doped boron nitride nanosheets toward toxic hydrogen fluoride (HF) gas. Molecular dynamics simulations conclusively validate the structural integrity of doped nanosheets. The adsorption energy calculations demonstrated that HF binds most strongly to pristine BN (−2.42 eV), followed by P‐BN (−2.28 eV), with the weakest binding on Al‐BN (−1.28 eV), indicating that all interactions are classified as chemisorption. However, Al‐BN exhibited the fastest recovery time at just 3.28 × 10^−2^ s at 500 K with ultraviolet irradiation. HF adsorption‐induced changes in the bandgap and work function revealed enhanced electrical conductivity. Optical properties, including the absorption coefficient and reflectivity, display pronounced UV‐spectrum peaks. This detailed analysis reveals that P‐doped BN is the most sensitive and selective candidate for detecting HF. Meanwhile, Al‐doped BN offers rapid desorption and operational adaptability, establishing the foundation for designing top‐performing sensors.

## Graphical abstract

This study reveals how pristine, P‐doped, and Al‐doped boron nitride nanosheets interact with toxic hydrogen fluoride (HF) gas using density functional theory (DFT) simulation. Enhanced adsorption and charge transfer in doped systems highlight their potential as sensitive and selective gas sensors, providing molecular‐level insights for nanoscale sensing applications.

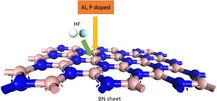

## 1 Introduction

Hydrogen fluoride (HF) is marked as a strongly penetrable gas that can act as a stimulant for the human body. The reaction between phosphate ore and sulfuric acid produces HF as a byproduct [1]. The gas consists of organic halogens, which are typically emitted into the atmosphere from both natural and anthropogenic sources at the Earth's surface [2]. This gas is most widely used in industry as a fluoride compound that is highly corrosive and reacts as an unstable liquid [3]. HF is a crucial industrial feedstock used in producing a wide spectrum of fluorine‐containing materials, including refrigerants, agrochemicals, pharmaceuticals, high‐octane gasoline, polymers, electronic devices, and fluorescent lighting. Due to its highly toxic properties, HF ranks at the top of the warning list among hazardous chemicals [4]. Exposure to HF concentrations above 30 ppm is acutely life‐threatening, while prolonged exposure to levels as low as 3 ppm can cause significant damage to bones and internal organs [5, 6]. It also affects the respiratory system and the skin of the human body, and also causes skeletal defects, pulmonary hemorrhage, dental fluorosis, bronchiolar ulceration, and cardiovascular problems [7, 8]. For protecting the environment and human health, unwanted HF must be removed.

Two‐dimensional (2D) nanomaterials are widely investigated for gas sensing materials because of their large specific surface area, high chemical reactivity, and outstanding carrier mobility [9, 10]. By the method of sensing hazardous gases, our environment will be saved from the toxicity of gases [11]. Boron nitride (BN) is a perfect gas‐sensing material, with a wide bandgap of ~6 eV, and also has specific optical, electronic, and magnetic properties like Coulomb blockade, super magnetism, and photoluminescence [12, 13]. Experimentally, the phase stability of BN under pressure is observed [14]. It is revealed that BNs exhibit limited sensitivity to gaseous molecules because of their tiny surface area [15]. Conversely, few‐layer BNs are promising candidates for high‐performance gas sensing applications. The alternation of boron (B) and nitrogen (N) atoms within BN layers gives rise to its ionic nature that may provide strong sensitivity toward different gaseous species. The adsorption of gas molecules, a necessity for a gas sensor, significantly alters the electrical characteristics of BNs. Additionally, the 2D structure of BN enables complete surface atom accessibility to adsorbing gases, leading to increased sensitivity. This material may be utilized in difficult environments where other materials cannot be used because of its great thermal stability and chemical inertness [16].

Among many types of 2D nanomaterials, BN nanosheets provided remarkable structural, electrical, mechanical, optical, chemical, and thermal properties in various applicable conditions [17, 18, 19, 20, 21, 22, 23]. Moreover, it has a good phase stability [24, 25] along with its graphene‐like structure, which offers superior thermal stability and chemical activity compared to graphene [26, 27, 28]. In contrast, BN nanosheets’ atomic layers demonstrate excellent gas‐sensing capabilities. Zeng et al. employed plasma etching to assess the conductivity of multiwalled BN nanoribbons experimentally. The study revealed that the conductivity of BN nanoribbons arises from vacancy defects and exposed edges [29]. BN nanosheets have obtainable piezo‐electric characteristics and dielectric phenomena [30]. The bandgap of structurally altered BN sheets was reduced, accompanied by magnetic characteristics. Their study reveals that it has the potential to detect toxic gas in critical environmental conditions [31]. Marjaneh Samadizadeh et al. demonstrated, via density functional theory, that BN nanosheets serve as effective sensors for toxic nitrous oxide [32]. Multiple investigations indicate that BN nanomaterials are promising for applications in gas storage, insulating lubricants, heat‐resistant semiconductors, and electronic devices [33, 34, 35]. Furthermore, doping BN with metal atoms can significantly enhance its reactivity and gas‐sensing capabilities [36, 37]. Researchers have also indicated that a Pd‐doped h‐BN monolayer could serve as an efficient nanomaterial for the detection of SF_6_ decomposition products [9]. Study shows BNNTs of appropriate diameters exhibit high stability and favorable physisorption toward HCHO, making them promising candidates for efficient and recyclable formaldehyde adsorption [38]. The adsorption behavior of NH_3_ on BNNTs is strongly influenced by tube diameter and structural defects, with deficient edges favoring chemisorption and diameter‐dependent effects on charge transfer and recovery time [39]. Another research reported a comparative density functional study on Pd‐ and Ni‐doped BN nanosheets, focusing on their surface reactivity and catalytic activity for NO reduction via CO [40]. Another study examined the stability and catalytic performance of h‐BN nanosheets doped with various metals (Cu, Ag, Au, Pt, Rh, Pd, Fe, Co, and Ir) for CO oxidation [41]. Also study shows, Si and Ge doped Graphene have been shown to improve hydrogen adsorption and activation on nanomaterials, which is efficient for applications in energy conversion and storage [42]. Besides, a study reported that the sensing of toxic CO gas on pristine, Co‐doped, and P‐doped BN nanosheets revealed that Co‐doped BNNS exhibits the strongest adsorption, the largest decrease in the HOMO‐LUMO gap, and the shortest nonbonding adsorption distance (AD), indicating its superior potential for CO detection [43]. According to Derdare et al., density functional theory (DFT) calculations show that yttrium‐ and zirconium‐doped BN nanosheets exhibit enhanced structural stability, electronic properties, and adsorption performance toward formamide, retaining high sensitivity and ultrafast recovery in aqueous environments, indicating their potential as reusable, high‐performance gas sensors [44].

Here, in this article, we designed and optimized the BN and doped BN nanosheet using DFT. We explored the structural, electronic, optical, adsorption, and electrical properties of the BN nanosheet to understand the behavior of the interaction between the gas nanosheets. In this study, we investigated theHF gas sensitivity of both pure and P‐ and Al‐doped BN nanosheets by using DFT. As far as we know, there is still no work between these two dopant systems, as well as with these gases.

## 2 Computational Details

All calculations were performed within the plane‐wave pseudopotential framework (Ultrasoft Vanderbilt type) based on DFT, as implemented in the Cambridge Serial Total Energy Package (CASTEP) [45]. The interaction of HF gas with pristine, P‐doped, and Al‐doped BN nanosheets was investigated using density functional theory. The generalized gradient approximation (GGA), specifically the Perdew Burke Ernzerhof (PBE) functional, was applied to define electron exchange and correlation in the property analysis [46] along with the implementation of ultrasoft pseudopotentials. To ensure better accuracy, a 3 × 2 × 1 BN nanosheet supercell was constructed, and all calculations employed a 3 × 3 × 1 k‐point mesh within the Monkhorst–Pack scheme [45]. We use a cutoff energy of 500 eV for all calculations. Throughout the study, Grimme's dispersion correction method was used to account for long‐range van der Waals interactions [47]. Furthermore, the most stable adsorption configuration was determined with the “Adsorption Locator Tool” in Materials Studio. A single N atom (B site) in the middle of the supercell is replaced with an impurity P atom to form a P‐doped BN nanosheet. Similarly, the supercell formed an Al‐doped BN nanosheet by replacing the B atom. To examine the time‐dependent stability of the nanosheets, molecular dynamics (MD) simulations were conducted using Forcite. The simulations ran for 10 ps at 298 K under the microcanonical (NVE) ensemble [48]. To better understand how the adsorbents and adsorbates interact, we used reduced density gradient (RDG) analysis, carried out with the help of VMD and Multiwfn software [49, 50]. The sensitivity of the nanosheets toward toxic gas adsorption was examined by calculating the adsorption energy ($E_{ads}$) using the formula below [51, 52].

(1) $$E_{\mathrm{ads}}=E_{\mathrm{NS}+\mathrm{gas}}-E_{\mathrm{NS}}-E_{\mathrm{gas}}$$

where $E_{\mathrm{NS}+\mathrm{gas}}$ corresponds to the energy of the nanosheet with the adsorbed gas, $E_{\mathrm{NS}}$ to the pristine nanosheet, and $E_{\mathrm{gas}}$ to the isolated gas molecule.

## 3 Results and Discussion

### 3.1 Geometry Analysis

**FIGURE 1:**
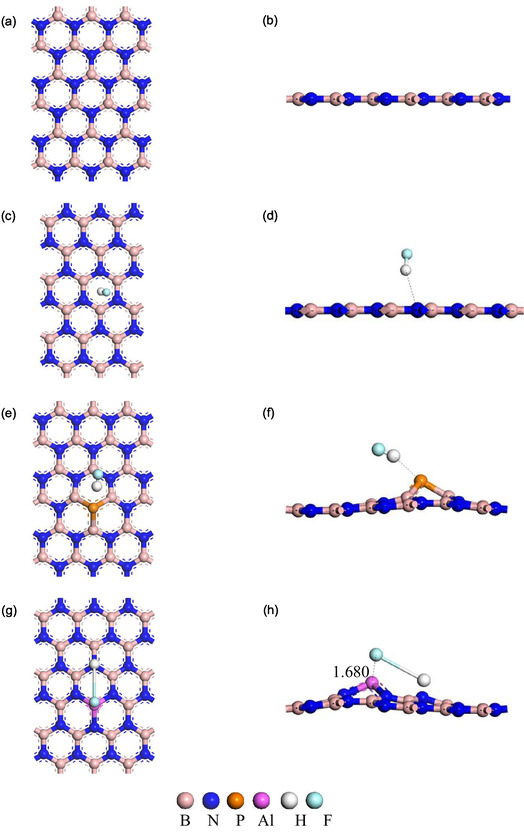
The optimized structure for (a, b) BN, (c, d) BN + HF, (e, f) P‐BN + HF, and (g, h) Al‐BN + HF nanosheets in front and side views, respectively.

Understanding structural stability is crucial for any material. In this study, we studied the geometrical structures of BN, P‐BN, and Al‐BN nanosheets before and after interaction with different gas molecules (Figure 1). In the optimized BN nanosheet, the total number of atoms is 36, where the number of boron (B) atoms is 18, and the number of nitrogen (N) atoms is 18. The calculated B–N bond length (1.449 Å) is in excellent agreement with the experimentally implied value for hexagonal BN (~1.44–1.45 Å) and previously reported DFT results (1.44–1.46 Å). This confirms the reliability of the adopted computational approach [53, 54]. We further examined P‐doped and Al‐doped BN nanosheets, obtained by replacing a nitrogen atom with P and a boron atom with Al in the pristine structure, to enhance stability and adsorption capability. The nanosheets’ cohesive energy was determined according to the equation below [55],

(2) $$E_{\text{cohesive}}=\frac{1}{n}[E_{\text{nanosheet}}-X_{B}E_{B}-Y_{N}E_{N}]$$

where *n* denotes the total number of atoms in the nanosheet, while $X_{B}$, $Y_{N}$ represent the number of boron and nitrogen atoms, respectively; $E_{\text{nanosheet}}$ is the total energy of the BN nanosheet; and $E_{B}$, $E_{N}$ correspond to the energies of isolated boron and nitrogen atoms. The BN, P‐BN, and Al‐BN nanosheets’ cohesive energies are −8.82, −8.66, and −8.57 eV/atom, respectively, and they are all negative and considerable, as in the previous research [56], supporting the stability of the adsorbents. Hence, these nanosheet structures are stable, suggesting potential for the synthesis of toxic gases within the nanosheets. From cohesive energy calculations, it is evident that the pristine BN nanosheet is the most stable among the three structures. We investigated the optimized geometries of BN, P‐BN, and Al‐BN nanosheets, together with their corresponding gas adsorbed configurations. The optimized structure of both P‐BN and Al‐BN nanosheets reveals slight structural distortions compared to pristine BN.

After optimizing the geometries of BN, P‐BN, and Al‐BN, the average bond lengths of B–N, B–P, and Al–N were calculated (Table 1), showing strong agreement with earlier reports [43, 56, 57, 58, 59, 60]. In this research, we measured the bond length for the gas and sheet. The measured bond lengths are shown in Table 1. As shown in the table, HF adsorption leads to slight variations in bond length for BN and P‐BN, but a pronounced change is evident for Al‐BN.

**TABLE 1:** Bond lengths for adsorbents and their gas adsorbed configurations.

Bond type	BN, Å	BN + HF, Å	P‐BN, Å	P‐BN + HF, Å	Al‐BN, Å	Al‐BN + HF, Å
B‐N	1.449	1.450	1.457	1.460	1.475	1.456
B‐P	—	—	1.702	1.882	—	—
Al‐N	—	—	—	—	1.696	1.813
H‐F	—	0.953	—	0.968	—	3.204

### 3.2 Molecular Dynamics (MD)

**FIGURE 2:**
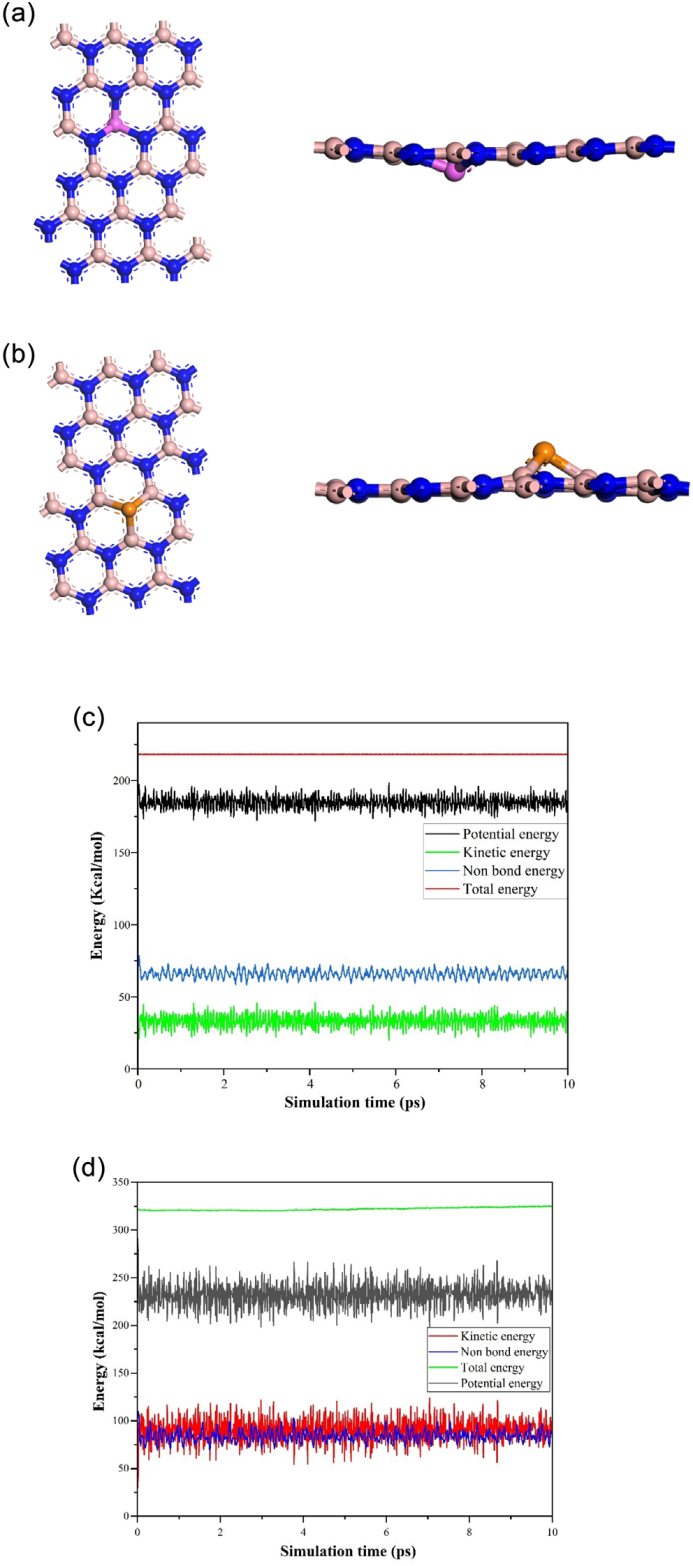
Structure of (a) Al‐BN, (b) P‐BN after molecular dynamics (MD) simulation. The energy (Kcal/mol) versus simulation time (ps) graph of (c) Al‐BN, and (d) P‐BN.

This analysis examines the MD simulation. Figure 2 results for Al and P‐doped BN systems, with a priority on their energy stability and fluctuations throughout a 10 ps simulation. For Al‐BN, the energy exhibits fluctuations limited to a narrow range of 1 kcal/mol, and the minor energy variation of approximately 0.46% suggests a system of high stability throughout the simulation [61]. The consistently stable total energy indicates effective energy conservation with negligible drift, which is a characteristic feature of a dependable MD simulation. In the case of P‐BN about a 1.5% variation concerning the average value. The variation is greater than that observed in the Al‐doped system, yet it remains within the acceptable thresholds for MD simulations [62]. In both simulations, the total energy stays almost constant (flat line), which is optimal for NVE MD.

### 3.3 Adsorption Energy Analysis

Adsorption energy is defined as the interaction energy required for gas molecules to bind to the surface of an adsorbent. We have calculated the adsorption energy for HF gas using Equation (1), which is shown in Table 2. Also measured the corresponding AD [63, 64]. The HF gas shows negative adsorption energy with pure and doped sheets. The negative adsorption energy signifies a direct interaction between the gas molecules and the underlying base layers [65]. The adsorption energies are comparable to HF adsorption on graphene [8] and penta‐PdAs_2_ monolayer [66]. The observed similarity in adsorption energy and AD highlights the capability of the nanosheets as efficient adsorbents. Generally, an adsorption energy less than −1 eV is classified as physisorption, while values greater than −1 eV indicate chemisorption [67]. Therefore, the adsorption of HF gas on BNs and doped BNs is characterized as chemisorption. Higher adsorption means that smaller quantities of adsorbent are sufficient to achieve the desired removal rates, which helps lower operational and material costs over time [68]. HF is a polar molecule; this orientation is consistent with the charge distribution of both the molecule and the surface. The nitrogen atoms in the BN sheet carry a partial negative charge, while the hydrogen atom in HF is partially positive due to the high electronegativity of fluorine. As a result, an attractive electrostatic interaction forms between the H atom and the nitrogen sites of the sheet [8].

**TABLE 2:** Adsorption energy, adsorption distance (AD), and recovery time across various frequencies and temperatures.

Structures	$E_{\mathrm{ads}}$, eV	AD, Å	Temperature, 298K	Recovery time (s) ($f_{0}$= 3 × 10^14^ Hz)	Temperature, 500K	Recovery time (s) ($f_{0}$= 3 × 10^14^ Hz)
			Recovery time (s) ($f_{0}$ = 10^12^ Hz)		Recovery time (s) ($f_{0}$ = 10^12^ Hz)	
BN + HF	−2.42	2.57	3.09 × 10^29^ s	8.67 × 10^26^ s	3.03 × 10^12^	1.01 × 10^10^
P‐BN + HF	−2.28	2.20	3.03 × 10^26^ s	1.36 × 10^24^ s	1.03 × 10^11^	3.43 × 10^8^
Al‐BN + HF	−1.28	1.68	1.84 × 10^9^ s	5.8 × 10^6^ s	9.83	3.28 × 10^−2^

Materials with abundant adsorption sites may saturate or degrade quickly, particularly under harsh or cyclical conditions. This can affect their long‐term performance and lead to higher replacement costs [69]. Both phosphorus and aluminum doping appear to reduce the stabilization energy (indicated by less negative values) when compared to pure BN. However, both dopants also lead to a shortening of the AD, which might suggest alterations in the bonding interactions or the way the molecules are adsorbed.

Introducing Al into the BN lattice leads to distortions in the structure and creates less optimal electronic interactions, which weaken the binding energy [27]. Al doping leads to the weakest adsorption (E_ads_ = −1.2890 eV), making it about 47% less favorable than pure BN and 44% weaker than P‐doped BN. Research indicates that the Al–N and Al–B bonds are generally weaker (−1.28 eV) than the native B–N bonds found in pure BN, and also weaker than the P–B bonds formed when P is used for doping [70]. The distance between H and F atoms in the Al‐doped BN system, where the HF molecule undergoes dissociative chemisorption rather than simple molecular adsorption. As documented in literature, HF molecules can dissociate on Al‐containing surfaces (such as Al‐doped graphene or AlN nanotubes), with the H and F atoms bonding to separate surface sites. This dissociation leads to a significant increase in the H–F distance, effectively breaking the intramolecular bond and confirming a strong chemisorption mechanism [71, 72]. Phosphorus, with its similar size and higher electronegativity compared to Al, can more easily integrate into the BN structure and form relatively stronger bonds (−2.28 eV) [73]. This order (BN > P‐BN > Al‐BN) suggests that dopant choice is a key factor in determining surface reactivity.

### 3.4 Recovery Time

A sensor's performance is evaluated based on its recovery time (τ), which is the desorption time of gas molecules from the nanosheet surface [74]. The recovery time (*τ*), a fundamental parameter of sensing materials, can be estimated using Equation (3),

(3) $$\tau =\frac{1}{f_{0}}e^{-E_{\mathrm{Ads}/\mathrm{KT}}}$$

where *K* and *T* are denoted as the Boltzmann constant (8.61 × 10^−5^ eV K^−1^) and temperature, respectively. The sensor can be experimentally restored by UV radiation at ($f_{0}$ = 10^12^ to 3 × 10^14^ Hz) at 298 K. In this study, recovery times were determined at two temperatures: *T* = 298 K and *T* = 500 K. Hence, we determined the recovery time *τ* using Equation (3) for different frequencies. At 298 K, the desorption times for BN and P‐BN are exceptionally long, ranging from 10^26^ to 10^29^ s, implying very strong adsorption and extremely slow desorption. In contrast, for Al‐BN, the recovery time is significantly shorter, around 10^9^ s, suggesting that desorption occurs more easily. Strong binding, when used in catalysis or sensing, results in increased stability and sensitivity. Longer molecular adsorptive times result in higher catalytic efficiency or detection limits. Excessively lengthy recovery periods make it difficult to practically regenerate or reuse the material. High temperatures or prolonged periods of time may be necessary to desorb adsorbates, which would raise operating costs and slow down sensor response times. All structures’ recovery periods are significantly shortened by a substantial factor when the temperature is raised to 500 K because of improved molecular mobility and desorption kinetics. The recovery period for Al‐BN at 500 K is exceedingly short (~10 s or less), indicating very limited HF retention and quick desorption. The system is better suited for repetitive sensing applications or catalysis, where rapid turnover is required, particularly at moderate temperatures, and recovery durations are faster. The practical limits of slow desorption at room temperature are overcome by increasing the temperature, which exponentially shortens recovery time. The regeneration of adsorbents after gas adsorption is typically achieved through chemical treatment or intense electromagnetic radiation. In cases involving weak to moderate interactions between gas molecules, ultraviolet (UV) light can effectively facilitate recovery. Several commonly used regeneration agents, including HCl, NaOH, HNO_3_, and NaHCO_3_, might be more successful in restoring both adsorbents [75].

### 3.5 Electronic Properties Analysis

To investigate the electronic state and behavior of electrons in BN, P‐BN, and Al‐BN nanosheets before and after selective gas adsorption, we examined various electronic properties, including band structures, Hirshfeld charges, Mulliken charges, and density of states (DOS).

#### 3.5.1 Band Structure Analysis

**FIGURE 3:**
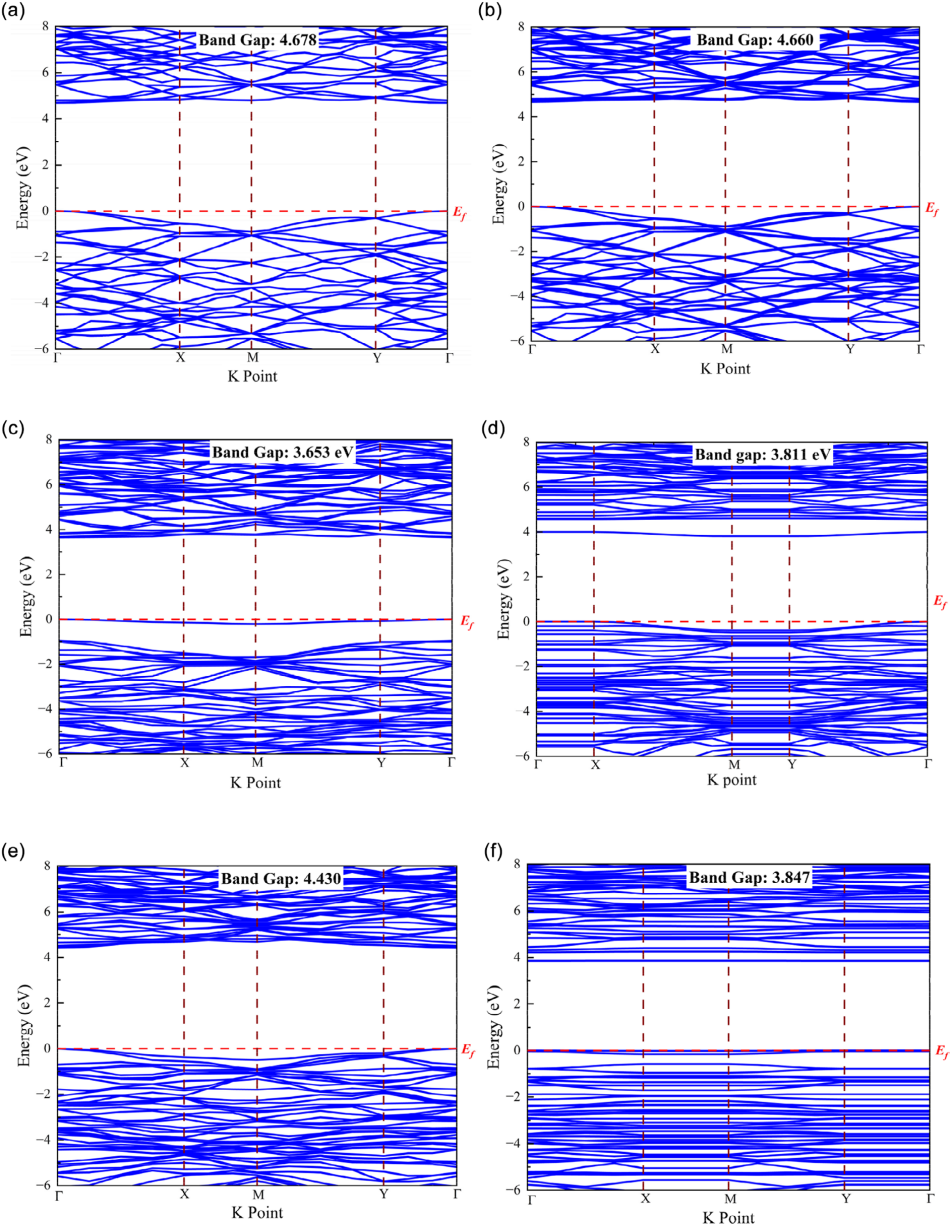
Band structures of (a) BN, (b) HF + BN, (c) P‐BN, (d) HF + P‐BN, (e) Al‐BN, and (f) HF + Al‐BN.

The energy interval between the conduction band and the valence band is termed the bandgap [76]. Figure 3 shows the band structures of the adsorbent and gas‐adsorbed systems. The calculated band structures reveal direct bandgaps, where the conduction band minimum (CBM) and valence band maximum (VBM) occur at the same K point. Figure 3a,c and e, demonstrate that the pure BN, P‐BN, and Al‐BN single layers indeed have a bandgap (Table 3) of 4.678, 3.653, and 4.430 eV, which is comparable to earlier research [77, 78, 79, 80]. The presence of phosphorus significantly lowers the bandgap to 3.653 eV, enhancing the material's conductivity and responsiveness in lower energy (longer wavelength) ranges. This indicates improved capabilities for applications in electronics and optoelectronics [81]. Al doping lowers the bandgap relative to pure BN (4.678 eV → 4.430 eV) but less than P doping (4.430 eV vs. 3.653 eV). Aluminum, classified as a group III element, possibly behaves as an acceptor, creating states close to the valence band. We observe variations in the bandgap, both increasing and decreasing, following gas adsorption. This suggests that the electron structure undergoes alterations following its interaction with the gas. HF gas molecules either donate electrons or accept them from the host material. Charge transfer may induce the formation of impurity states within the bandgap, which can narrow or widen the bandgap by altering the distance between the valence and conduction bands [82]. The strong bonding of HF gas with the nanosheet often leads to hybridization between its molecular orbitals and the electronic states of the host material [83]. This may alter band edges or create states within the initial bandgap. Through the adsorption of HF gas on P‐BN, the bandgap is slightly increased, but the bandgap is decreased through the adsorption of HF gas on Al‐BN nanosheet. Figure 3 presents a detailed illustration of the electronic band structure (EBS) series of energy levels for the adsorbent and adsorption system, mapped along high‐symmetry points in the sequence Γ–X–M–Y–Γ. Alterations in the bandgap following gas adsorption led to tangible advantages in sensor sensitivity, selectivity, signal strength, and the overall functionality of materials. This provides the framework for advanced gas sensors, environmental monitoring systems, and optoelectronic devices [84].

**TABLE 3:** Bandgap for adsorbents and their gas adsorbed configurations.

Structure	Bandgap, eV
BN	4.678
BN + HF	4.660
P‐BN	3.653
P‐BN + HF	3.811
Al‐BN	4.430
Al‐BN + HF	3.847

#### 3.5.2 Hirshfeld Charges Analysis

Hirshfeld charge analysis is a computational method employed to evaluate the distribution of electronic charge across individual atoms within a molecule. The concept focuses on deformation density, wherein the overall electron density of a molecule is analyzed by comparing it with the electron densities of separate atoms in their isolated, neutral configurations [85]. As presented in Table 4, the Hirshfeld charge analysis reveals that the boron (B) atom possesses a positive charge, whereas the nitrogen (N) atom exhibits a negative charge. Compared to the Mulliken charge scheme, the Hirshfeld method yields slightly different values for partial positive and negative charges due to changes in computational methodology. Nevertheless, both methods reflect a consistent trend in charge polarity. Upon adsorption of HF gas onto the BNs, P‐BNs, and Al‐BNs nanosheets, the Hirshfeld charges exhibit slight changes. Changes in both Hirshfeld and Mulliken charge distributions after gas adsorption provide evidence of notable interactions between the nanosheets and the gas species.

**TABLE 4:** Average Hirshfeld charges of structural elements expressed in,e| units.

Elements	BN	BN + HF	P‐BN	P‐BN + HF	Al‐BN	Al‐BN + HF
B	0.21	0.21	0.196	0.204	0.209	0.214
N	−0.21	−0.21	−0.206	−0.21	−0.227	−0.218
P	—	—	0.00	0.00	—	—
Al	—	—	—	—	0.54	0.44
H	—	0.16	—	0.11	—	0.16
F	—	−0.21	—	−0.22	—	−0.31

#### 3.5.3 Mulliken Charges Analysis

The Mulliken charge analysis reveals the nature of bonding characteristics between atoms. By examining the charge distribution from atom to atom, it is possible to determine whether a bond is covalent or ionic [76]. Higher charge sharing between atoms typically indicates covalent bonding, whereas lower charge interactions are characteristic of ionic bonding [86]. In our research, we investigated the charge transfer between gas molecules and nanosheets using Mulliken population analysis. In BN, P‐BN, and Al‐BN nanosheets, N atoms are electronegative, and B atoms are electropositive. B consistently exhibits a positive charge even after dope, indicating a continual loss of electrons, thereby working as an electron donor. Following the adsorption of HF (i.e., BN + HF), there is a slight decrease in the charge on boron (Table 5), suggesting a minor reduction in electron loss or a shift in electron distribution upon adsorption. N exhibits a persistent negative charge because of its electronegativity, indicating its ability to acquire electrons from B, dopants, or the HF gases. P in P‐BN possesses a slight positive charge (+0.30 e), yet following HF treatment, it approaches a neutral state. This indicates that P operates as an electron donor and is diminished or considerably impacted by HF adsorption, implying a direct interaction between P and HF or a substantial alteration of the surface environment. Al exhibits a notably high positive charge (2.24 e and 2.15 e), indicating substantial electron donation, surpassing that of B or P. The adsorption of HF results in a slight decrease in the positive charge of Al, indicating a minor redistribution of electrons or a weak interaction with F or H compared to other structures. The variation in charge transfer modifies the electronic structure of the material, consequently affecting its measurable electrical signal. This makes the Mulliken analysis an easy measure of sensor performance [87, 88].

**FIGURE 4:**
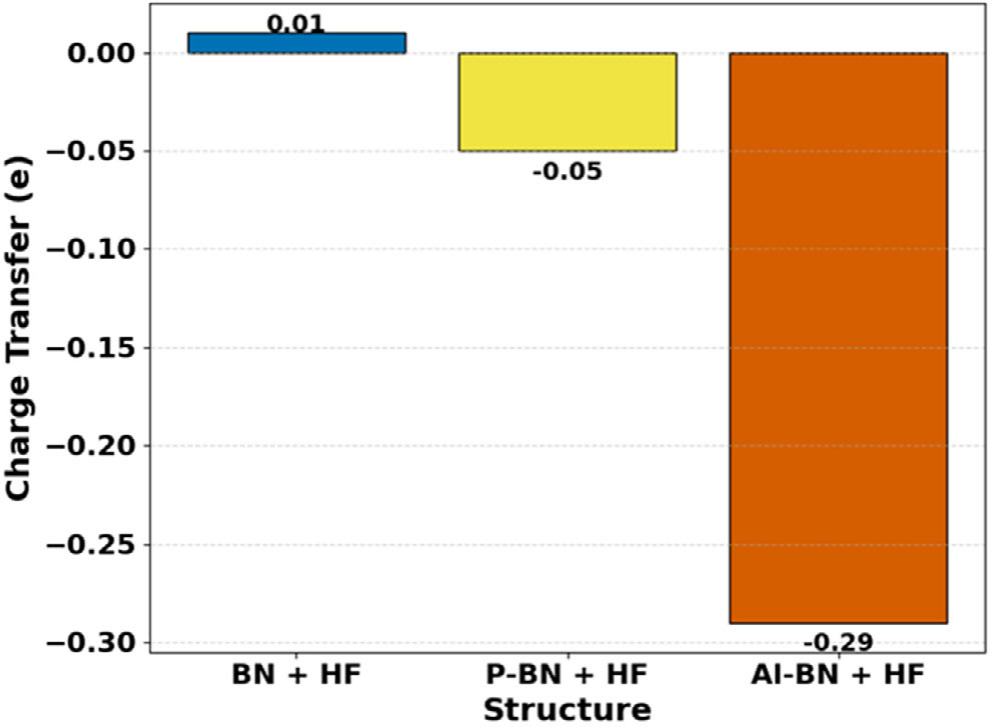
Charge transfer analysis between gas molecules and adsorbent surfaces upon adsorption.

The interaction of the adsorbent with the adsorbate is provided by the charge transfer data presented in Figure 4. Positive charge transfer values denote electron flow from the gas molecule to the nanosheet, while negative values represent electron transfer in the opposite direction [63].

**TABLE 5:** Average Mulliken charges of structural elements expressed in,e| units.

Elements	BN	BN + HF	P‐BN	P‐BN + HF	Al‐BN	Al‐BN + HF
B	0.84	0.834	0.771	0.709	0.80	0.812
N	−0.84	−0.836	−0.835	−0.828	−0.879	‐ 0.869
P	—	—	0.30	−0.00	—	—
Al	—	—	—	—	2.24	2.15
H	—	0.62	—	0.57	—	0.44
F	—	−0.61	—	−0.62	—	−0.73

#### 3.5.4 Electron Density Difference (EDD)

**FIGURE 5:**
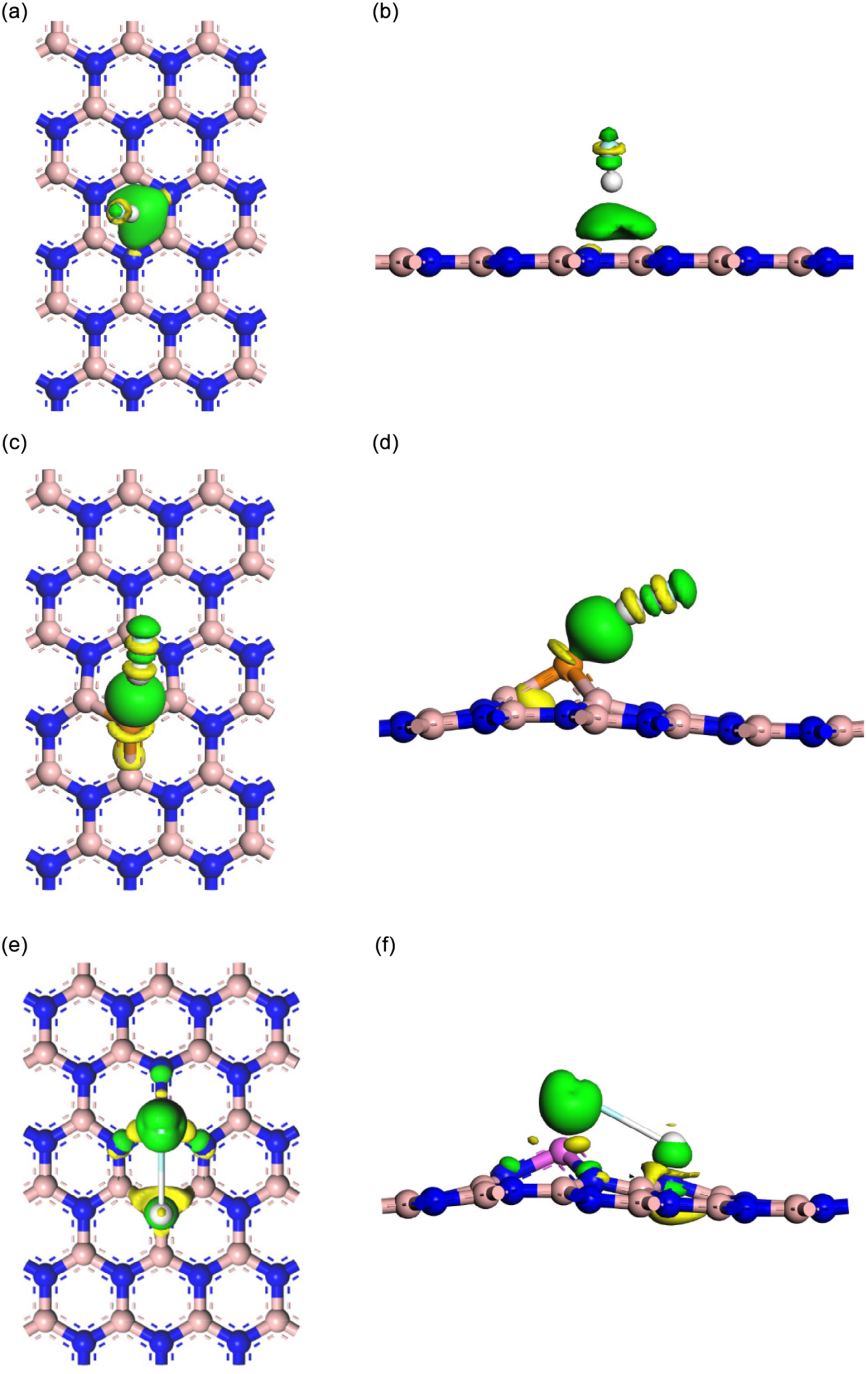
EDD on the top view and the side view of (a) BN, (b) HF + BN, (c) P‐BN, (d) HF + P‐BN, (e) Al‐BN, and (f) HF + Al‐BN.

Figure 5 illustrates the electron density difference (EDD) for the nanosheets with adsorbed HF gas, shown at the top and side views, respectively. In these visualizations, green areas indicate regions of high electron density (electron‐rich), while yellow areas highlight regions with significant electron deficiency (electron‐depletion), suggesting potential sites for electron gain from the green regions. However, doping BNs with P and Al introduced notable changes in both the electronic structure and adsorption energy. The relatively high electronegativity of fluorine in HF, when compared to boron and nitrogen, facilitates the transfer of electrons from BN to HF. This results in an accumulation of electrons near fluorine and a depletion near boron. The redistribution of electron density can enhance the reactivity of BN surfaces, making them valuable as catalysts or sensors for polar molecules such as HF [89]. Wider and more pronounced green and yellow areas near the adsorption site for P‐BN + HF, Figure 5c,d. The regions of both accumulation and depletion became larger in comparison to pristine BN. The presence of P enhances the local electronic activity; the P site exhibits increased chemical reactivity due to its higher lone pair character. The findings indicate enhanced charge transfer, along with increased polarization and deformation of the electron cloud [73]. The analysis of both top (plan view) and side views, Figure 5e,f for Al‐BN + HF, reveals a significant redistribution of multiple atoms surrounding the Al‐doped site. Al works as an electron donor or an active site, greatly improving charge transfer effectiveness. Suggests a significantly enhanced interaction with HF, potentially addressing chemisorption instead of physisorption. This observation, enhanced sensitivity, is attributed to the formation of localized electronic states and charge redistribution upon doping, which facilitates greater interaction with the HF molecules. Increased EDD elevate reactivity with polar gases such as HF, which is advantageous for gas detection and catalytic processes.

#### 3.5.5 Density of States (DOS)

**FIGURE 6:**
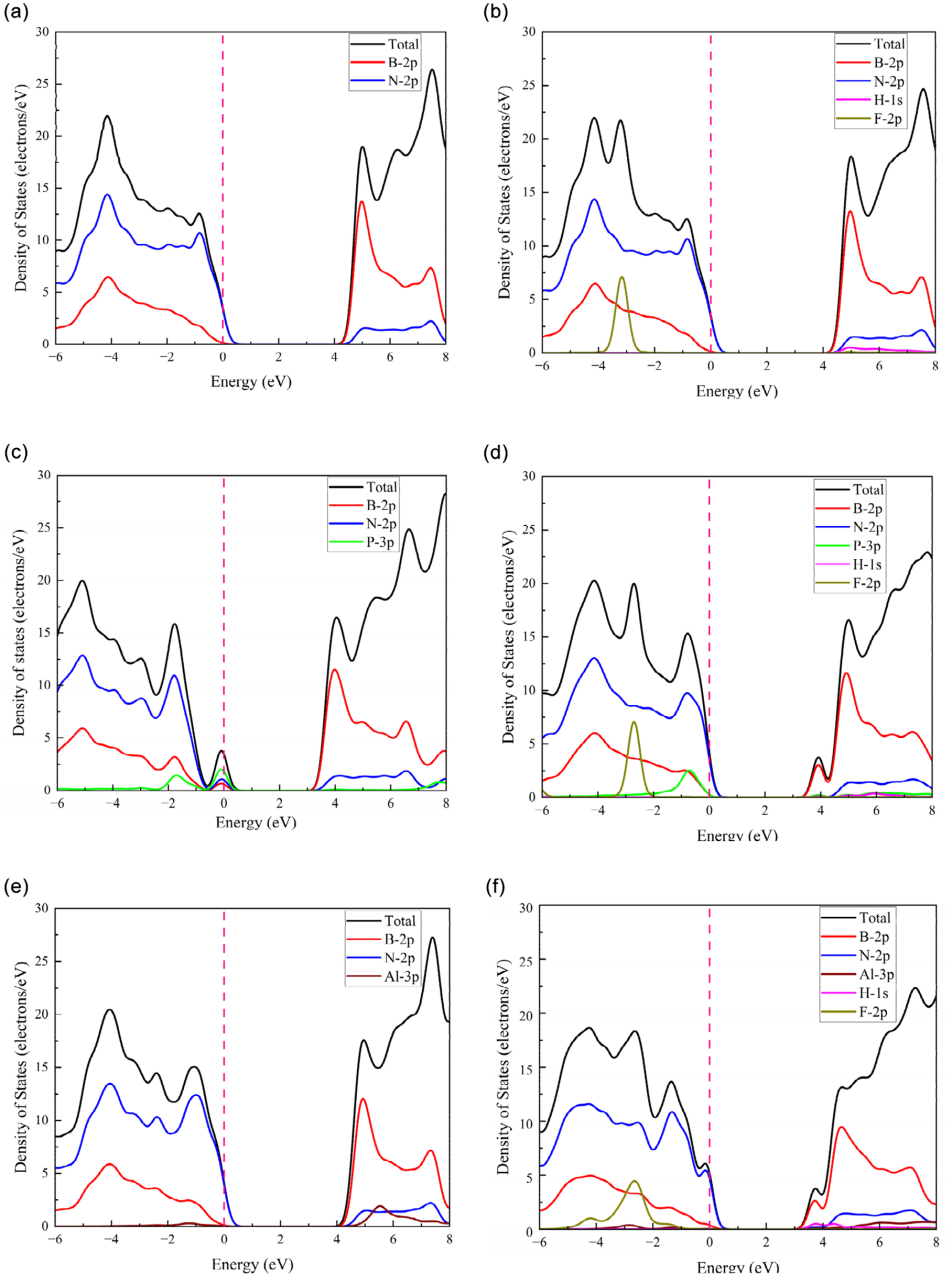
Density of states of (a) BN, (b) HF + BN, (c) P‐BN, (d) HF + P‐BN, (e) Al‐BN, and (f) HF + Al‐BN.

The DOS is the numerical calculation of energy surface integrals within the Brillouin zone [90]. Generally, DOS refers to the distribution of available electron states per unit energy and unit volume across different energy levels. The DOS analysis provides insight into the electronic nature of a material, distinguishing between insulator, semiconductor, and metallic characteristics. When no energy states are present at the Fermi level, a bandgap is formed, implying that the material behaves as a semiconductor or an insulator [91]. The total and partial DOS (TDOS and PDOS) of the optimized structures, both before and after adsorption, are analyzed and presented in Figure 6.

Figure 6 illustrates the TDOS and PDOS for the BNs, P‐BNs, and Al‐BNs. However, after the adsorption of HF gas onto the sheets, notable changes occur in the VB and CB peaks. Our analysis of BNs reveals that B‐2p and N‐2p orbital electrons are the primary contributors to both the valence and conduction bands, as indicated by the PDOS. In the BNs + HF system, F‐2p orbital electrons also play a significant role in these bands, surpassing the contribution from H‐1s orbitals. This suggests stronger adsorption of HF gas on BNs and all doped nanosheets, with pronounced hybridization implying the formation of stronger chemical bonds. However, the structures demonstrate strong interactions with the investigated gas molecules, as indicated by the electronic responses observed in the DOS and PDOS analyses. The DOS analysis reveals that doping BN nanosheets with elements like P and Al significantly alters the electronic structure. These modifications, especially the introduction of states near the Fermi level, can enhance electrical conductivity and gas adsorption properties, making doped BN nanosheets promising candidates for sensor applications.

#### 3.5.6 Conductivity

Electrical conductivity (*σ*) in materials is significantly influenced by the bandgap, which governs the thermal excitation and availability of charge carriers [92]. The following equation describes the relationship between electrical conductivity, bandgap energy, and temperature in semiconductor materials [63]

(4) $$\sigma =Aexp\left(\frac{-E_{g}}{2kT}\right)$$

The constant A remains unknown due to the lack of experimental synthesis of these structures. Therefore, only the exponential term shows how electrical conductivity changes with the bandgap energy. The conductivity σ is expressed in units of A× Ω^−1^ m^−1^. The calculated conductivity values for the studied structures are summarized in Table 6. This study reveals that a reduction in bandgap leads to a significant exponential increase in electrical conductivity, which also shows a positive trend with increasing temperature from 300 to 500 K. The electrical conductivity of pristine BN was calculated to be 5.07 × 10^−40^ A× Ω^−1^ m^−1^. Upon HF adsorption, the conductivity increased by approximately 41.61% at 300 K compared to that of pristine BN. In contrast, the conductivity of P‐BN was calculated as 2.07 × 10^−31^ A× Ω^−1^ m^−1^, which decreased to 9.72 × 10^−33^ A× Ω^−1^ m^−1^ after HF adsorption. For the Al‐BN + HF complex, the conductivity increased significantly to 4.85 × 10^−33^ A× Ω^−1^ m^−1^, indicating a substantial enhancement. We observed that the electrical conductivity of P‐BN upon HF adsorption is nearly twice that of Al‐BN, representing an increase of approximately 100%.

**TABLE 6:** Summary of Fermi energy, work function (*ϕ*), bandgap (eV), and electrical conductivity (*σ*) of adsorbents before and after gas adsorption at varying temperatures from 300 to 500 K.

Structure	Fermi energy, eV	ϕ, eV	∇, ϕ	Bandgap, eV	Conductivity, A × Ω^−1^m^−1^		
					300 K	400 K	500 K
BN	−3.610	3.6102	—	4.678	5.07 × 10^−40^	3.38 × 10^−30^	2.65 × 10^−24^
BN + HF	−1.934	1.9348	1.6754	4.660	7.18 × 10^−40^	4.39 × 10^−30^	3.26 × 10^−24^
P‐BN	−1.412	1.4120	—	3.653	2.07 × 10^−31^	9.69 × 10^−24^	3.88 × 10^−19^
P‐BN + HF	−1.048	1.0486	0.3634	3.811	9.72 × 10^−33^	9.79 × 10^−25^	6.20 × 10^−20^
Al‐BN	−1.311	1.3112	—	4.430	6.14 × 10^−38^	1.23 × 10^−28^	4.71 × 10^−23^
Al‐BN + HF	−3.528	3.5281	2.2169	3.847	4.85 × 10^−33^	5.81 × 10^−25^	4.09 × 10^−20^

#### 3.5.7 Work Function

Work function (*ϕ*) refers to the least amount of energy needed for an electron to escape from the Fermi level of a material into the vacuum [93]. It can be calculated using the following formula [94],

(5) $$\phi =|V_{\infty }-E_{F}|$$

**FIGURE 7:**
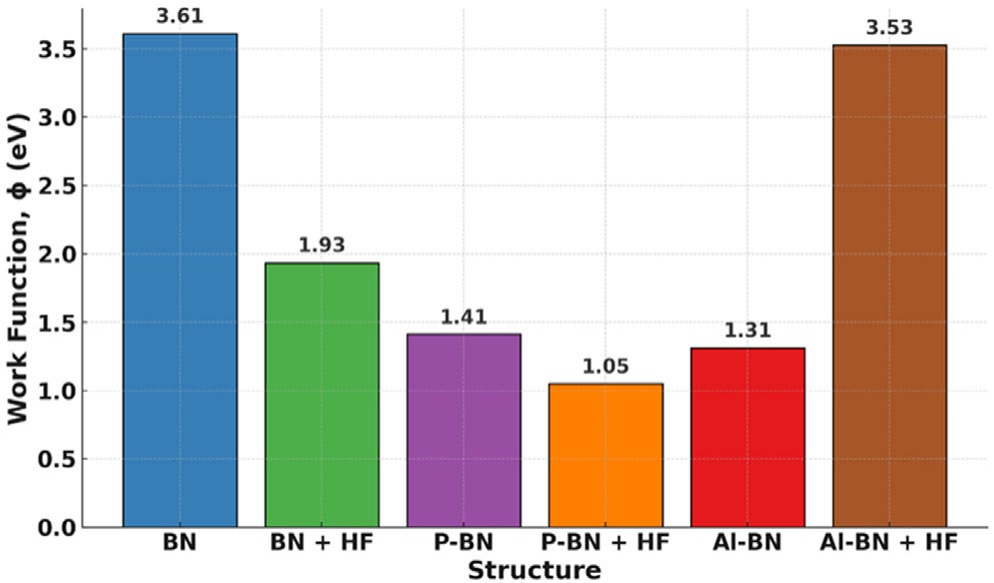
Variation in the work function of pristine and doped BN caused by HF adsorption, illustrating the influence of surface interactions.

The term $V_{\infty }$ refers to the vacuum potential measured at an infinite distance from the adsorbent surface, where it is conventionally taken as zero, and $E_{F}$ represents the Fermi energy [95]. A summary of the calculated work function values for the various configurations is presented in Table 6. The effects of HF gas adsorption on the work function of pristine and modified BN are illustrated in Figure 7. In this study, gas adsorption resulted in a decrease in the work function values for BN and P‐BN complexes, whereas an increase was observed for Al‐BN. These variations suggest charge transfer interactions between the adsorbent and the adsorbed gas molecules. The initial work function of pristine BN (3.6102 eV) decreased by nearly 46.4% following HF adsorption, yielding a value of 1.9348 eV. The P‐BN + HF system exhibited the lowest work function (1.0486 eV) among the gas‐adsorbed configurations, indicating favorable electron emission characteristics. On the other hand, Al‐BN + HF exhibited the highest work function value (3.5281 eV), indicating suppressed electron emission and suggesting its potential use as a protective material. We also observed that the work function of pristine BN decreased by approximately 60.9% and 63.7% upon doping with P and Al, respectively. These results suggest enhanced electronic interactions and superior sensing performance. The work function undergoes notable changes upon gas adsorption, measurable by the Kelvin method, providing a basis for work function‐based sensor development [96]. The Richardson–Dushman relation provides the mathematical framework describing the effect of work function on conductivity changes as follows [97],

(6) $$J_{R}=B_{R}T^{2}e^{-\frac{\phi }{k_{B}T}}$$

where $B_{R}$ is the Richardson constant and $J_{R}$ is the current density of emitted carriers. A reduction in the work function ϕ results in an exponential increase in carrier emission, highlighting the sensitivity of this process to changes in ϕ.

#### 3.5.8 Sensitivity

To explore the interaction mechanism, we analyzed the sensitivity of the complexes across the 300–500 K temperature range. The sensitivity was obtained using the following equation [98]:

(7) $$S=e^{\frac{\left|\Delta E_{\mathrm{gap}}\right|}{2k_{B}T}}-1$$

where $\Delta E_{\mathrm{gap}}$ corresponds to the change in bandgap between the nanosheet after gas adsorption and its pristine form. $k_{B}$ represents the Boltzmann constant (8.62 × 10^−5^ eV/K, and T denotes the absolute temperature [99]. Table 7 illustrates the variation in sensitivity of BN, P‐BN, and Al‐BN toward HF gas across different temperatures. In this study, a gradual decrease in sensitivity was observed across all investigated structures as the temperature increased. This trend arises from the diminished interaction between gas molecules and the surface at higher temperatures. Al‐BN exhibits superior sensitivity to the HF gas molecule at 300 K compared to all other studied structures. Conversely, BN shows the lowest sensitivity toward HF, and its sensitivity decreases steadily with increasing temperature. Additionally, P‐BP demonstrates the second greatest sensitivity to HF compared to the other structures. The sensitivity of P‐BN decreases by approximately 56.2% at 400 K and 74.1% at 500 K as the temperature increases from 300 to 500 K. These findings demonstrate that Al‐doped BN exhibits significantly enhanced sensitivity toward HF gas molecules compared to pristine BN, with the sensitivity trend following Al‐BN > P‐BN > BN.

**TABLE 7:** Comparison of sensitivities for various gas‐adsorbed structures at 300, 400, and 500 K.

Structure	Sensitivity		
	300 K	400 K	500 K
BN + HF	0.416	0.298	0.232
P‐BN + HF	20.26	8.883	5.253
Al‐BN + HF	78,969	4712.5	867.5

### 3.6 Optical Properties Analysis

The optical properties of a material determine its interaction with light. In this study, we investigated various optical parameters, including absorption coefficient (AC), reflectivity, and conductivity for BNs, P‐BNs, and Al‐BNs nanosheets, before and after gas adsorption.

#### 3.6.1 Absorption Coefficient (AC)

The AC indicates the depth to which light of a specific wavelength can penetrate a material before being absorbed. The AC in the UV region is on the order of 10^5^ to 10^6^ cm^−1^, indicating strong absorption near the band edge.

**FIGURE 8:**
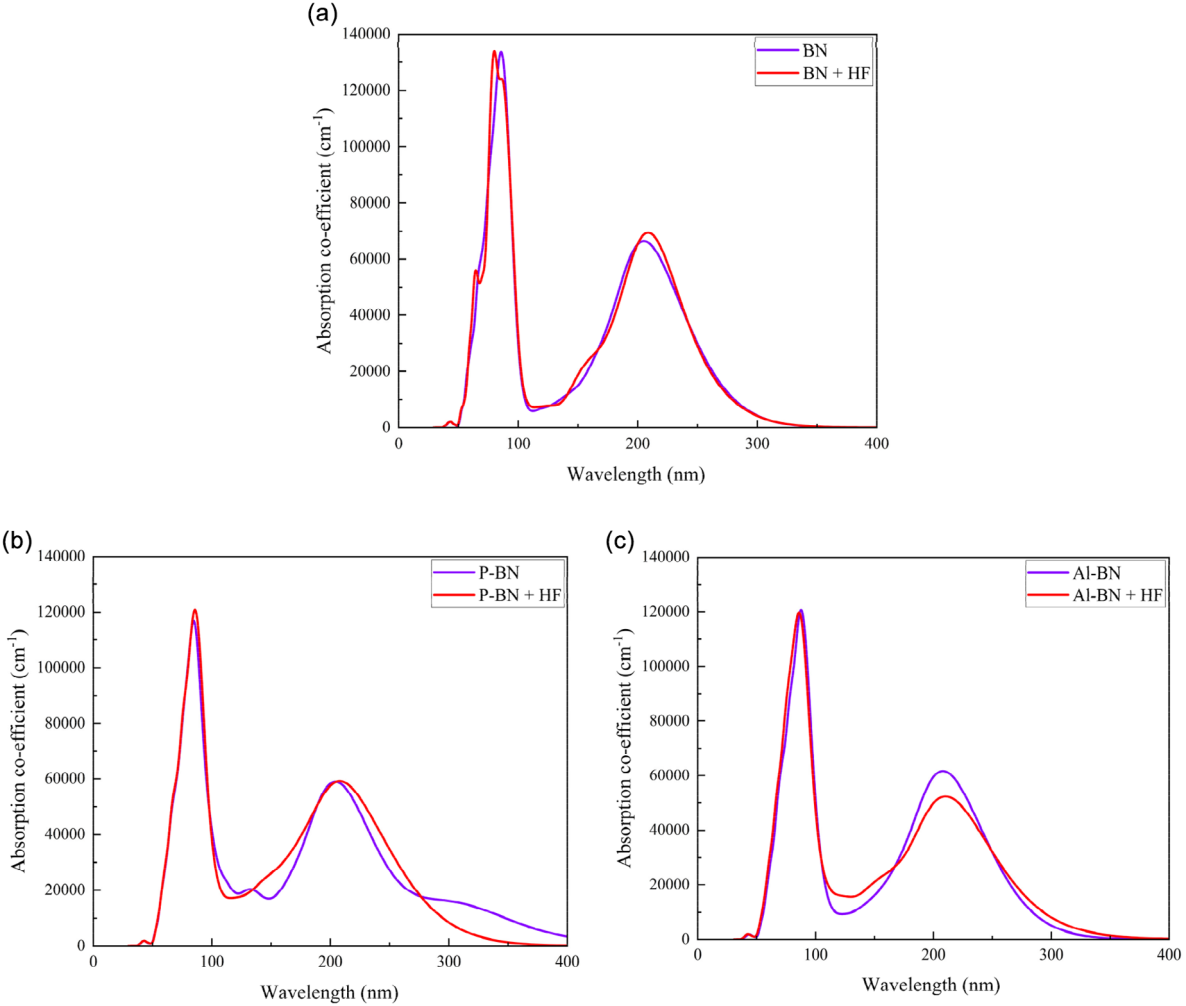
Absorption coefficient for (a) BN, (b) P‐BN, and (c) Al‐BN nanosheets with HF gas.

The AC of BN nanosheets varies based on factors such as their structural form, the number of layers, and the wavelength of the incoming light. Figure 8 illustrates the AC for BNs, P‐BNs, and Al‐BNs both before and after gas adsorption. All the structures exhibit a similar AC profile, showing a peak in the ultraviolet energy range, particularly below 200 nm.

The highest peak among all structures appears around 50–150 nm. After absorbing the gas, we noted both redshifts. The threshold point in absorption refers to the minimum energy a material begins to absorb from electromagnetic radiation. In the case of a BN nanosheet, this threshold occurs around 80 nm, whereas for P‐BN and Al‐BN nanosheets, it shifts to approximately 85 nm. The presence of phosphorus and aluminum alters the BN lattice, creating new energy levels that engage with HF, resulting in a shift and enhancement of absorption peaks. The optical detection exhibits both selectivity and sensitivity, highlighted by the distinct comparison observed before and after gas exposure [100].

#### 3.6.2 Reflectivity Analysis

**FIGURE 9:**
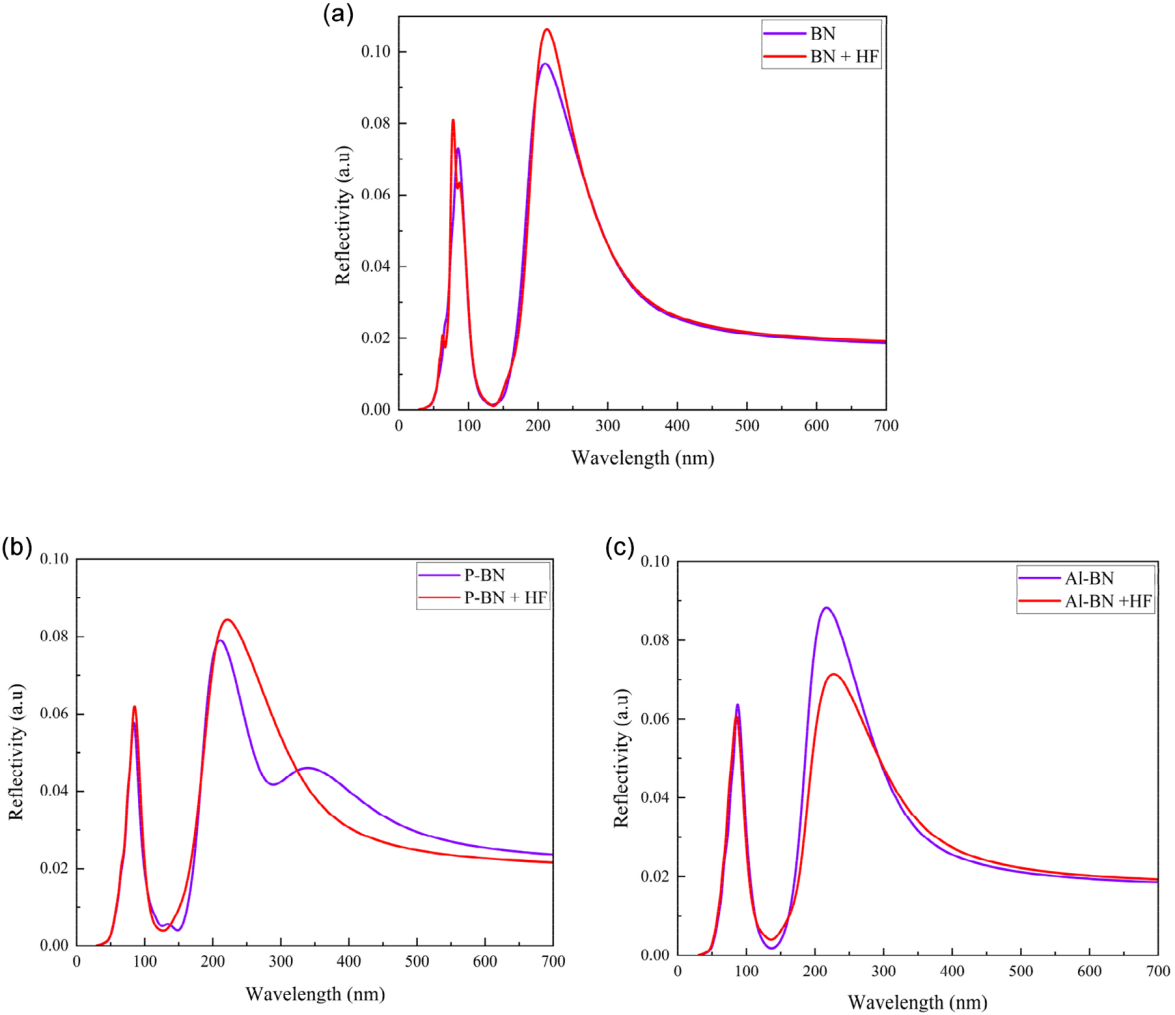
Reflectivity for (a) BN, (b) P‐BN, and (c) Al‐BN nanosheets with HF gas.

Figure 9 represents the calculated optical reflectivity of BN, P‐BN, and Al‐BN nanosheets exposed to toxic HF gas as a function of wavelength. Before and after gas absorption, all structure shows high reflectivity in the UV‐wavelength region. The Al‐BN + HF shows lower (7%) reflectivity, Figure 9c, compared to the others. Alterations in electronic properties, such as those induced by doping, photoexcitation, or laser irradiation, can lead to an increase in absorption or a shift in transition thresholds, resulting in reduced reflectivity [101]. Devices such as solar cells and photodetectors gain significant advantages from reduced reflection, which enhances light absorption and conversion efficiency.

After gas adsorption, the reflectivity spectra exhibit slight shifts in peak positions and changes in peak intensity without Al‐BN. As a result, the type of adsorbed gas can be identified based on the reflected energy.

#### 3.6.3 Conductivity Analysis

Optical conductivity describes how a material responds to an alternating electromagnetic field and is directly related to its ability to absorb and transport energy via electrons. It is commonly derived from absorption data and is especially useful in analyzing 2D materials like BN nanosheets.

**FIGURE 10:**
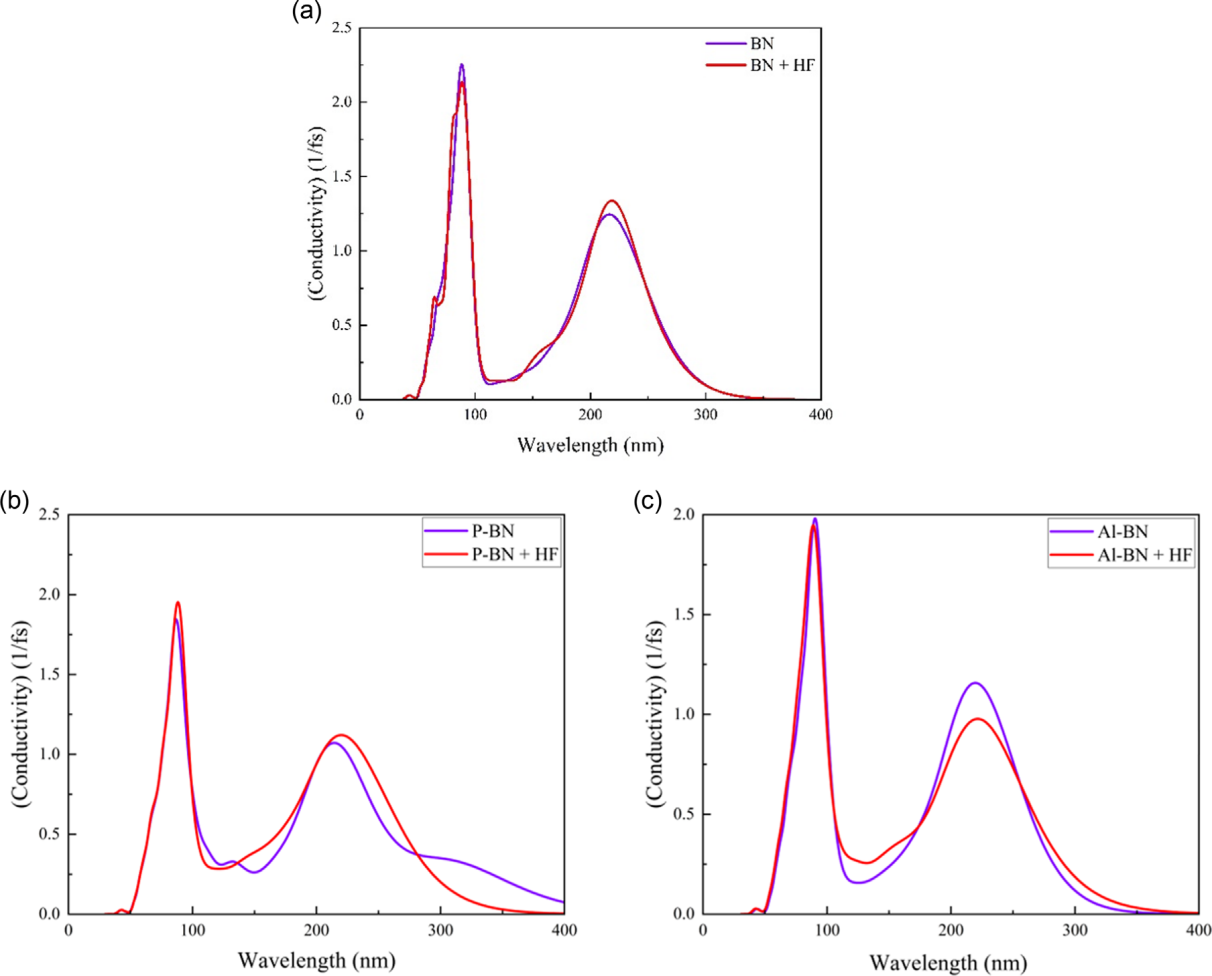
Conductivity for (a) BN, (b) P‐BN, and (c) Al‐BN nanosheets with HF gas.

Figure 10 illustrates the conductivity behavior of BNs, P‐BNs, and Al‐BNs nanosheets before and after exposure to toxic gases. The highest conductivity in BNs was observed in the UV range. Following the HF gas adsorption of BNs, both P‐BNs and Al‐BNs exhibit approximately similar conductivity peaks. The highest conductivity was observed in the pristine BN and its complex, Figure 10a. Figure 10b shows that the conductivity of P‐BN peaks at a wavelength of almost 95 nm, both before and after gas adsorption. Beyond this wavelength, the conductivity gradually decreases after 220 nm. In Figure 10c, introducing gases to Al‐BNs slightly reduces the peak, with the UV range showing the highest conductivity.

### 3.7 RDG Analysis

The RDG approach enables the spatial visualization of noncovalent interactions by analyzing the electron density and its gradient within molecular systems [92]. It is mathematically represented by the following expression [97]:

(8) $$\mathrm{RDG}(r)=c\frac{|\nabla \rho (r)|}{\rho {\left(r\right)}^{4/3}}$$

**FIGURE 11:**
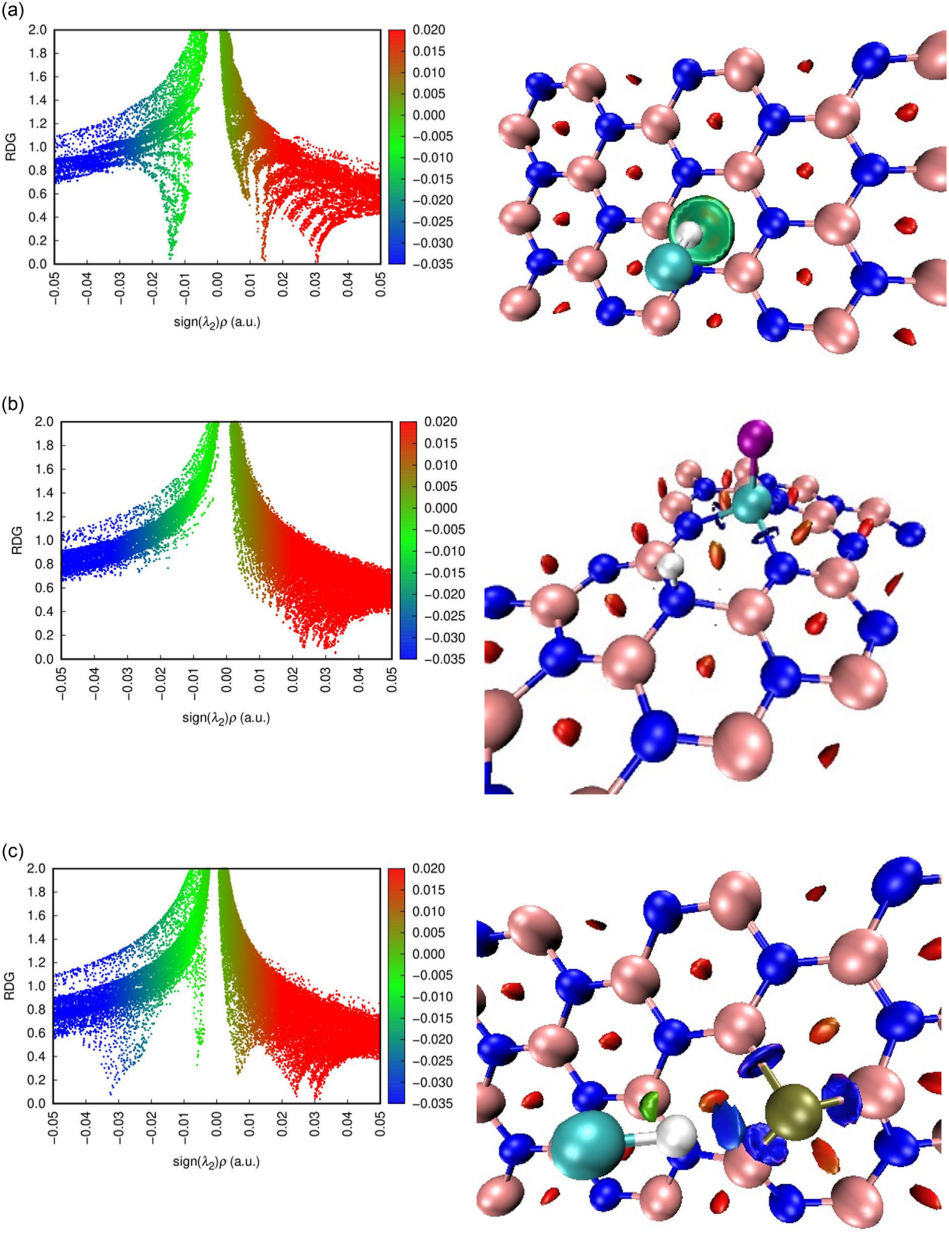
Visualization of RDG scatter plot and isosurfaces for (a) BN + HF, (b) Al‐BN + HF, and (c) P‐BN + HF complexes.

In this expression, ρ(r) denotes the electron density at a point r, ∇ρ(r) signifies the rate of change of the density, and c is a constant of proportionality [97]. The RDG scatterplots and isosurface plots for pristine and metal‐doped BN in interaction with the HF toxic gas molecule are shown in Figure 11. The vertical axis of the scatterplot represents RDG, while the horizontal axis corresponds to the product of the electron density and the sign of the second eigenvalue of the Hessian matrix, denoted as sign (λ_2_)ρ. Low electron density regions suggest weak van der Waals forces, whereas high‐density areas with sharp gradients indicate stronger noncovalent interactions involving significant electronic contributions [102]. The sign(λ_2_)ρ value facilitates the interpretation of interaction behavior, with positive values (>0) indicating repulsive interactions, negative values (<0) suggesting attractive interactions, and values near zero (≈0) corresponding to weak interactions [103]. In Figure 11, sign(λ_2_)ρ values, ranging from −0.035 to 0.020 a.u., are shown through a blue‐to‐red color gradient. For the interaction between pristine BN and HF, the RDG isosurfaces appear green, indicating regions where the second eigenvalue of the electron density Hessian (λ_2_) approaches zero, which is characteristic of weak van der Waals interactions. The results suggest that the interaction is dominated by physisorption, exhibiting negligible charge transfer. Following Al doping, distinct blue isosurfaces are observed between HF and Al sites (λ_2_ < 0), indicative of attractive interactions like hydrogen bonding. The adsorption of HF on Al‐BN reveals significantly stronger interaction regions, reflected by more intense blue‐green isosurfaces (λ_2_ values typically between −0.03 and −0.04 a.u.) compared to pristine BN. The H–F bond breaks upon adsorption, and both F and H atoms bind to the Al‐doped BN nanosheet. This occurs because Al substitution creates a highly electropositive active site that strongly interacts with the electronegative F atom, inducing charge transfer and weakening the H–F bond [104, 105, 106]. This observation implies a higher binding affinity, likely driven by orbital hybridization or electron donation induced by the presence of the Al atom. In the P‐BN + HF system, the RDG isosurfaces exhibit noticeably increased intensity and extend over a significantly larger area of the BN surface. Furthermore, the isosurfaces display distinct and pronounced blue spikes, indicating stronger attractive interactions, with λ_2_ values closely clustering near −0.03 a.u.

### 3.8 Discussion on Adsorption and Sensing Mechanisms

The gas‐sensing effectiveness of pristine, P‐ and Al‐doped BN nanosheets arises from the integration between their structural, electronic, and optical properties. MD simulations and cohesive energy calculations confirm the structural stability of the doped systems, ensuring they can withstand interactions with HF gas. The sensing mechanism is driven by chemisorption, where strong adsorption energies (−01.28 to −2.42 eV) induce significant electronic reconfiguration. P‐BN exhibits the highest sensitivity due to substantial reductions in work function and bandgap, which enhance electrical conductivity upon gas exposure. A trade‐off between sensitivity and recovery time is observed: P‐BN shows the strongest electronic response but slower recovery, whereas Al‐BN, with its higher charge transfer, provides faster recovery (9.83 s at 500 K), making it suitable for real‐time applications. Moreover, notable shifts in optical absorption and reflectivity offer an additional UV‐based detection modality. These analyses collectively demonstrate that P‐doping maximizes sensitivity, while Al‐doping optimizes the sensor for rapid, repetitive use Table 8.

### 3.9 Comparison Table

**TABLE 8:** Comparative DFT analysis of HF adsorption on various pristine and doped 2D nanosheets, including adsorption energy (*E*_ads_), bandgap (*E*_g_), charge transfer (Δ*q*), and work function (*Φ*).

Structure	***E***_**ads**_ ,**eV**	*E*_g_ ,eV	Δ*q* ,e	Φ ,eV	Ref.
BN	−2.42	4.660	0.01	1.68	This work
P‐BN	−2.28	3.811	−0.05	0.36	This work
Al‐BN	−1.28	3.847	−0.29	2.22	This work
ZnS ML	−0.86	2.94	−0.08	6.402	[107]
CuCl ML	−0.31	3.56	−0.04	4.74	[108]
Ti‐doped hBN	−0.81	0 (metallic)	−0.10	4.91	[109]
Au‐MoTe_2_	−0.23	1.035	−0.033	—	[110]
Ag‐MoTe_2_	−0.32	1.034	−0.007	—	[110]
Cu‐MoTe_2_	−0.33	1.115	+ 0.031	—	[110]
penta‐PdAs_2_ ML	−0.39	0.30	+ 0.111	4.708	[111]
BP	−0.276	0.879	−0.08	1.719	[92]
Al‐BP	−1.651	1.007	−0.62	1.576	[92]
Ti‐BP	−0.502	0.000	−0.06	1.643	[92]

## 4 Conclusion

This study provides a thorough theoretical study of how pristine BN nanosheets and nanosheets doped with different elements adsorb toxic HF gas using density functional theory calculations. Analyses of cohesive energies show that the nanosheets are thermodynamically stable; MD simulations further support the stability of these materials. HF adsorption energy values show that the interaction strength follows BN, suggesting chemisorption in all cases. BN + HF shows an extremely long recovery time of 3.09 × 10^29^ s at 298 K (for 10^12^ Hz UV), and Al‐BN + HF exhibits a drastically shorter time of 9.83 s. Electronic structure analyses demonstrate that doping raises the bandgap, with values of 3.811 eV for phosphorus‐doped BN and 3.847 eV for aluminum‐dopedBN. Optical properties indicate that all of the studied materials primarily absorb ultraviolet light, and that doping induces modifications to absorption and reflectivity profiles following gas exposure. This study highlights how doping BN with specific elements can significantly enhance its ability to detect hazardous HF gas with greater sensitivity, selectivity, and speed—offering a promising step toward safer environmental practices and more reliable industrial monitoring.

## Author Contributions

**Nihal Siddique**: data curation, formal analysis, investigation, visualization, writing – original draft. **Rashek Dewan Daymond**: data curation, formal analysis, investigation, visualization, writing – original draft. **Md. Hasan Shahria Fahim**: data curation, formal analysis, investigation, visualization, writing – original draft. **Utso Jyoti Golder‐**visualization, validation, writing – original draft. **Debashis Roy**: formal analysis, project administration, validation, writing – review & editing. **Abdullah Al Roman**: resources, software, validation, writing – review & editing. **Mohammad Tanvir Ahmed**: conceptualization, methodology, project administration, supervision, writing – review & editing.

## Conflicts of Interest

The authors declare no conflicts of interest.

## Data Availability

The data supporting the findings of this study are not publicly available, as this research is part of ongoing work. The input files used for these calculations are available from the author upon reasonable request.

## References

[1] T. Dahlke , O. Ruffiner , and R. Cant , “Production of HF from H_2_SiF_6_ .“Procedia Engineering 138 (2016): 231–239, 10.1016/J.PROENG.2016.02.080.

[2] M. D. Cheng , “Atmospheric Chemistry of Hydrogen Fluoride,” Journal of Atmospheric Chemistry 75, no. 1 (2018): 1–16, 10.1007/S10874-017-9359-7.

[3] G. L. Waldbott and J. R. Lee , “Toxicity from Repeated Low‐Grade Exposure to Hydrogen Fluoride—Case Report,” Clinical Toxicology 13, no. 3 (1978): 391–402, 10.3109/15563657808988247. 743869

[4] H. T. Kwak , H. Kim , H. Yoo , et al., “Hydrogen Fluoride Gas Sensor by Silicon Nanosheet Field‐Effect Transistor,” IEEE Sensors Journal 23, no. 15 (2023): 16545–16552, 10.1109/JSEN.2023.3285892.

[5] J. E. Colman Lerner , E. Y. Sanchez , J. E. Sambeth , and A. A. Porta , “Characterization and Health Risk Assessment of VOCs in Occupational Environments in Buenos Aires, Argentina,” Atmospheric Environment 55 (2012): 440–447, 10.1016/J.ATMOSENV.2012.03.041.

[6] H. Kabir , A. K. Gupta , and S. Tripathy , “Fluoride and Human Health: Systematic Appraisal of Sources, Exposures, Metabolism, and Toxicity,” Critical Reviews in Environmental Science and Technology 50, no. 11 (2020): 1116–1193, 10.1080/10643389.2019.1647028.

[7] H. Lim , K. Um , and S. Jung , “A Study on Effective Mitigation System for Accidental Toxic Gas Releases,” Journal of Loss Prevention in the Process Industries 49 (2017): 636–644, 10.1016/j.jlp.2017.05.017.

[8] D. Ghosh , G. Periyasamy , and S. K. Pati , “Adsorption of HF Pollutant on Single Vacant 2D Nanosheets: Ab Initio Molecular Dynamics Study,” Journal of Physical Chemistry C 117, no. 42 (2013): 21700–21705, 10.1021/jp407851z.

[9] S. Ma , D. Li , X. Rao , X. Xia , Y. Su , and Y. Lu , “Pd‐Doped h‐BN Monolayer: A Promising Gas Scavenger for SF_6_ Insulation Devices,“Adsorption 26, no. 4 (2020): 619–626, 10.1007/S10450-020-00226-3

[10] H. Cui , X. Zhang , J. Zhang , and Y. Zhang , “Nanomaterials‐Based Gas Sensors of SF_6_ Decomposed Species for Evaluating the Operation Status of High‐Voltage Insulation Devices,” High Voltage 4, no. 4 (2019): 242–258, 10.1049/hve.2019.0130.

[11] H. Nazemi , A. Joseph , J. Park , and A. Emadi , Advanced Micro‐and Nano‐Gas Sensor Technology: A Review, Sensors (Switzerland). MDPI AG, 2019, 10.3390/s19061285. PMC6470538 30875734

[12] X. F. Jiang , Q. Weng , X. Bin Wang , et al., “Recent Progress on Fabrications and Applications of Boron Nitride Nanomaterials,” A Review. Journal of Materials Science and Technology 31, no. 6 (2015): 589–598, 10.1016/j.jmst.2014.12.008.

[13] T. Oku , A. Nishiwaki , and I. Narita , “Formation and Atomic Structure of B_12_N_12_ Nanocage Clusters Studied by Mass Spectrometry and Cluster Calculation,” Science and Technology of Advanced Materials 5, no. 5‐6 (2004): 635–638, 10.1016/j.stam.2004.03.017.

[14] E. Kim and C. Chen , “First‐Principles Study of Phase Stability of BN under Pressure,” Physics Letters A 319, no. 3–4 (2003): 384–389, 10.1016/j.physleta.2003.10.052.

[15] M. Sajjad and P. Feng , “Study the Gas Sensing Properties of Boron Nitride Nanosheets,” Materials Research Bulletin 49, no. 1 (2014): 35–38, 10.1016/J.MATERRESBULL.2013.08.019.

[16] Z. Wang , M. J. Meziani , A. K. Patel , et al., “Boron Nitride Nanosheets from Different Preparations and Correlations with Their Material Properties,” Industrial and Engineering Chemistry Research 58, no. 40 (2019): 18644–18653, 10.1021/acs.iecr.9b03930.

[17] J. Wang , F. Ma , W. Liang , R. Wang , and M. Sun , “Optical, Photonic and Optoelectronic Properties of Graphene, h‐NB and Their Hybrid Materials,” Nanophotonics 6, no. 5 (2017): 943–976, 10.1515/nanoph-2017-0015 Walter de Gruyter GmbH.

[18] D. Roy , M. K. Hossain , S. M. Hasan , Milon , M. A. Hossain , and F. Ahmed , “Understanding the Atomistic Origin of the Magnetic Phases in Cobalt‐TM (V, Nb, Ta, Zr, Hf, W) Pair Co‐Doped Boron Nitride Monolayer and the Hydrogenation Effect,” Physica E: Low‐Dimensional Systems and Nanostructures 125 (2021): 114359, 10.1016/j.physe.2020.114359.

[19] J. Wang , F. Ma , W. Liang , and M. Sun , Electrical Properties and Applications of Graphene, Hexagonal Boron Nitride (h‐BN), and Graphene/h‐BN Heterostructures, Materials Today Physics (Elsevier Ltd., 2017), 6–34, 10.1016/j.mtphys.2017.07.001.

[20] R. Beiranvand and S. Valedbagi , “Electronic and Optical Properties of h‐BN Nanosheet: A First Principles Calculation,” Diamond and Related Materials 58 (2015): 190–195, 10.1016/j.diamond.2015.07.008.

[21] A. Falin , Q. Cai , E. J. G. Santos , et al., “Mechanical Properties of Atomically Thin Boron Nitride and the Role of Interlayer Interactions,” Nature Communications 8 (2017): 15815, 10.1038/ncomms15815. PMC5489686 28639613

[22] N. Kostoglou , K. Polychronopoulou , and C. Rebholz , “Thermal and Chemical Stability of Hexagonal Boron Nitride (h‐BN) Nanoplatelets,” Vacuum 112 (2015): 42–45, 10.1016/j.vacuum.2014.11.009.

[23] V. Salles , S. Bernard , R. Chiriac , and P. Miele , “Structural and Thermal Properties of Boron Nitride Nanoparticles,” Journal of the European Ceramic Society 32, no. 9 (2012): 1867–1871, 10.1016/j.jeurceramsoc.2011.09.002.

[24] A. Janotti , S. H. Wei , and D. J. Singh , “First‐Principles Study of the Stability of BN and C,” Physical Review B ‐ Condensed Matter and Materials Physics 64, no. 17 (2001): 4107, 10.1103/PhysRevB.64.174107.

[25] L. P. McDonnell , J. J. S. Viner , D. A. Ruiz‐Tijerina , et al., “Superposition of Intra‐ and Inter‐Layer Excitons in Twistronic MoSe_2_/WSe_2_ Bilayers Probed by Resonant Raman Scattering,” 2D Materials 8, no. 3 (2021): 035009, 10.1088/2053-1583/abe778.

[26] H. Wang , T. Taychatanapat , A. Hsu , et al., “BN/Graphene/BN Transistors for RF Applications. Ieeexplore.Ieee.OrgH,” T PalaciosIEEE Electron Device Letters 32, no. 9 (2011): 1209–1211, 10.1109/LED.2011.2160611 2011.

[27] G. Wang , K. Zheng , Y. Huang , et al., “An Investigation of the Positive Effects of Doping an Al Atom on the Adsorption of CO_2_ on BN Nanosheets: A DFT Study,” LQ TaoPhysical Chemistry Chemical Physics 22, no. 17 (2020): 9368–9374, 10.1039/D0CP00714E 2020Pubs.Rsc.OrgG. 32309825

[28] A. Nag , K. Raidongia , K. P. S. S. Hembram , R. Datta , U. V. Waghmare , and C. N. R. Rao , “Graphene Analogues of BN: Novel Synthesis and Properties,“ACS Publications 4, no. 3 (2010): 1539–1544, 10.1021/NN9018762. 20128601

[29] M. Amiri , J. Beheshtian , F. Shayeganfar , M. Faghihnasiri , R. Shahsavari , and A. Ramazani , “Electro‐Optical Properties of Monolayer and Bilayer Pentagonal BN: First Principles Study,” Nanomaterials 10, no. 3 (2020): 440, 10.3390/nano10030440. 32121427 PMC7153586

[30] K. H. Michel and B. Verberck , “Theory of Elastic and Piezoelectric Effects in Two‐Dimensional Hexagonal Boron Nitride,” Physical Review B ‐ Condensed Matter and Materials Physics 80, no. 22 (2009): 4301, 10.1103/PhysRevB.80.224301.

[31] Y. H. Zhang , K. G. Zhou , X. C. Gou , K. F. Xie , H. L. Zhang , and Y. Peng , “Effects of Dopant and Defect on the Adsorption of Carbon Monoxide on Graphitic Boron Nitride Sheet: A First‐Principles Study,” Chemical Physics Letters 484, no. 4–6 (2010): 266–270, 10.1016/j.cplett.2009.11.051.

[32] M. Samadizadeh , A. A. Peyghan , and S. F. Rastegar , “Sensing Behavior of BN Nanosheet toward Nitrous Oxide: A DFT Study,” Chinese Chemical Letters 26, no. 8 (2015): 1042–1045, 10.1016/j.cclet.2015.05.048.

[33] T. Alaa Hussein , N. M. Shiltagh , W. Kream Alaarage , R. R. Abbas , R. A. Jawad , and A. H. Abo Nasria , “Electronic and Optical Properties of the BN Bilayer as Gas Sensor for CO_2_, SO_2_, and NO_2_ Molecules: A DFT Study,” Results in Chemistry 5 (2023): 100978, 10.1016/j.rechem.2023.100978.

[34] X. Li , J. Zhao , and J. Yang , “Semihydrogenated BN Sheet: A Promising Visible‐Light Driven Photocatalyst for Water Splitting,” Scientific Reports 3 (2013): 1, 10.1038/srep01858. PMC3656388 23681171

[35] L. Hromadová and R. Martoňák , “Pressure‐Induced Structural Transitions in BN from Ab Initio Metadynamics,” Physical Review B ‐ Condensed Matter and Materials Physics 84, no. 22 (2011): 4108, 10.1103/PhysRevB.84.224108.

[36] Y. Huang , T. Yang , L. Yang , et al., “Graphene–boron Nitride Hybrid‐Supported Single Mo Atom Electrocatalysts for Efficient Nitrogen Reduction Reaction,” Journal of Materials Chemistry A 7, no. 25 (2019): 15173–15180, 10.1039/C9TA02947H.

[37] Y. G. Zhou , P. Yang , X. Sun , Z. G. Wang , X. T. Zu , and F. Gao , “First‐Principles Study of the Noble Metal‐Doped BN Layer,” Journal of Applied Physics 109, no. 8 (2011): 25, 10.1063/1.3569725.

[38] Z. Li , J. Li , S. Yang , and J. Yin , “Density Functional Theory Calculations on the Structures and Electronic Properties of Boron Nitride Clusters toward Formaldehyde,” Research on Chemical Intermediates 50, no. 1 (2024): 397–412, 10.1007/s11164-023-05184-3.

[39] J.‐C. Li , Z. Li , S. Yang , J. Yin , and Y. Hu , “Density Functional Theory Calculations on the Ammonia Interaction with BmNm (m = 47, 71, and 95) Tubular Clusters,” Structural Chemistry 36, no. 3 (2025): 1055–1066, 10.1007/s11224-024-02435-w.

[40] M. D. Esrafili , S. Heydari , and L. Dinparast , “A Comparative DFT Study About Surface Reactivity and Catalytic Activity of Pd‐and Ni‐Doped BN Nanosheets: NO Reduction by CO Molecule,“Structural Chemistry 30, no. 5 (2019): 1647–1657, 10.1007/S11224-019-01355-4

[41] S. Lin , X. Ye , R. S. Johnson , H. Guo , and X. Ye Lin , “First‐Principles Investigations of Metal (Cu, Ag, Au, Pt, Rh, Pd, Fe, Co, and Ir) Doped Hexagonal Boron Nitride Nanosheets: Stability and Catalysis of CO Oxidation,” The Journal of Physical Chemistry C 117, no. 33 (2013): 17319–17326, 10.1021/JP4055445, ACS Publications.

[42] M. Ghanbari , S. Afshari , and S. A. Nabavi Amri , “New Capability of Graphene as Hydrogen Storage by Si and/or Ge Doping: Density Functional Theory,” International Journal of Hydrogen Energy 45, no. 43 (2020): 23048–23055, 10.1016/j.ijhydene.2020.06.039.

[43] D. Milon Roy , and F. Ahmed , “A First Principle Investigation of the CO Gas Adsorption Property of Pristine, Cobalt (Co), and Phosphorus (P) Doped BN Nanosheets,” Physica B: Condensed Matter 680 (2024): 415839, 10.1016/j.physb.2024.415839.

[44] M. Derdare , A.‐G. Boudjahem , and N. Cheghib , “Y‐ and Zr‐Modified Boron Nitride Nanosheets as Efficient Sensors for Formamide: A First‐Principles Approach,” Journal of Molecular Graphics and Modelling 144 (2026): 109278, 10.1016/j.jmgm.2026.109278. 41534322

[45] K. Hossain , S. Khanom , F. Israt , M. K. Hossain , M. A. Hossain , and F. Ahmed , “First‐Principles Study on Structural, Mechanical and Optoelectronic Properties of Lead‐Free Mixed Ge–Sn Hybrid Organic‐Inorganic Perovskites,” Solid State Communications 320 (2020): 114024, 10.1016/j.ssc.2020.114024.

[46] J. P. Perdew , K. Burke , and Y. Wang , “Generalized Gradient Approximation for the Exchange‐Correlation Hole of a Many‐Electron System,” Physical Review B 54 (1996): 16533, 10.1103/PhysRevB.54.16533. 9985776

[47] R. González‐González , M. G. Salas‐Zepeda , and A. Tlahuice‐Flores , “New Two‐Dimensional Carbon Nitride Allotrope with 1 : 1 Stoichiometry Featuring Spine‐Like Structures: A Structural and Electronic DFT‐D Study,” Physical Chemistry Chemical Physics 21, no. 28 (2019): 15282–15285, 10.1039/C9CP02846C. 31268446

[48] A. M. Ali , M. Y. Kwaya , A. Mijinyawa , A. A. Aminu , and Z. M. Usman , “Molecular Dynamics and Energy Distribution of Methane Gas Adsorption in Shales,” Journal of Natural Gas Geoscience 8, no. 1 (2023): 1–15, 10.1016/J.JNGGS.2022.12.004.

[49] T. Lu , “A comprehensive Electron Wavefunction Analysis Toolbox for Chemists, Multiwfn.,” The Journal of Chemical Physics 161, no. 8 (2024): 709, 10.1063/5.0216272/3309709. 39189657

[50] T. Lu and F. Chen , “Multiwfn: A Multifunctional Wavefunction Analyzer,” Journal of Computational Chemistry 33, no. 5 (2012): 580–592, 10.1002/JCC.22885;CTYPE: STRING: JOURNAL. 22162017

[51] M. H. S. Fahim , S. R. Biswas , R. D. Daymond , et al., “T‐Boron‐Nitride and Black Phosphorus Nanoflakes for Anticancer Drug Delivery Application: A Computational Insight,” Journal of Molecular Liquids 438 (2025): 128799, 10.1016/j.molliq.2025.128799.

[52] A. A. Mehnaz , M. H. S. Fahim , D. Roy , A. Al Roman , and M. T. Ahmed , “Purification of Ni^2+^ Heavy Metal Ions from Wastewater Using γ‐Graphyne: Insights from DFT and QTAIM Analysis,” Solid State Communications 407 (2026): 116238, 10.1016/j.ssc.2025.116238.

[53] E. Nemati‐Kande , A. Pourasadi , F. Aghababaei , S. Baranipour , A. Mehdizadeh , and J. J. Sardroodi , “Quantum DFT Methods to Explore the Interaction of 1‐Adamantylamine with Pristine, and P, As, Al, and Ga Doped BN Nanotubes,” Scientific Reports 12, no. 1 (2022): 19972, 10.1038/s41598-022-24200-x. 36402905 PMC9675779

[54] A. Mahdizadeh , S. Farhadi , and A. Zabardasti , “Microwave‐Assisted Rapid Synthesis of Graphene‐Analogue Hexagonal Boron Nitride (h‐BN) Nanosheets and Their Application for the Ultrafast and Selective Adsorption of Cationic Dyes from Aqueous Solutions,” RSC Advances 7 (2017): 53984–53995, 10.1039/c7ra11248c.

[55] S. R. Biswas , M. H. S. Fahim , M. T. Ahmed , A. Al Roman , and D. Roy , “A First Principles Study of the Adsorption Behavior of H_2_S and SO_2_ Gases on AlN Nanosheets with Fluorine Defects,” Chemical Physics Letters 882 (2026): 142497, 10.1016/j.cplett.2025.142497.

[56] H. Milon , K. M., D. Roy , and F. Ahmed , “A First Principle Study to Investigate Structural, Electronic and Optical Properties of Pristine and Valency Comparable Co, P Decorated Graphene like Boron Nitride (BN) Nanosheets,” Phase Transitions 95, no. 12 (2022): 837–850, 10.1080/01411594.2022.2139699, REQUESTEDJOURNAL: JOURNAL: GPHT20;JOURNAL: JOURNAL: GPHT20;WGROUP: STRING: PUBLICATION.

[57] L. Y. Guo , S. Y. Xia , Y. Long , et al., “P‐Doped h‐BN Monolayer: A High‐Sensitivity SF6Decomposition Gases Sensor,” IEEE Sensors Journal 22, no. 19 (2022): 18281–18286, 10.1109/JSEN.2022.3193873.

[58] E. Nemati‐Kande , M. Abbasi , and M. D. Mohammadi , “DFT Studies on the Interactions of Pristine, Al and Ga‐Doped Boron Nitride Nanosheets with CH_3_X (X=F, Cl and Br,” Journal of Molecular Structure 1199 (2020): 126962, 10.1016/J.MOLSTRUC.2019.126962.

[59] D. G. Kvashnin , K. L. Firestein , Z. I. Popov , et al., “Al − BN Interaction in a High‐Strength Lightweight Al/BN Metal‐Matrix Composite: Theoretical Modelling and Experimental Verification,” Journal of Alloys and Compounds 782 (2019): 875–880, 10.1016/J.JALLCOM.2018.12.261.

[60] J. Beheshtian , H. Soleymanabadi , M. Kamfiroozi , and A. Ahmadi , “The H_2_ Dissociation on the BN, AlN, BP and AlP Nanotubes: A Comparative Study,” Journal of Molecular Modeling 18, no. 6 (2012): 2343–2348, 10.1007/S00894-011-1256-4. 21979405

[61] J. Fu , B. Wang , Y. Chen , et al., “Computational Analysis the Relationships of Energy and Mechanical Properties with Sensitivity for FOX‐7 Based PBXs via MD Simulation,” Royal Society Open Science 8, no. 2 (2021): 200345. 10.1098/RSOS.200345, PAGE: STRING: ARTICLE/CHAPTER. PMC8074742 33972835

[62] H. He , L. Li , R. Ya , et al., “Molecular Dynamics Simulation and Experimental Verification of the Effects of Vinyl Silicone Oil Viscosity on the Mechanical Properties of Silicone Rubber Foam,” RSC Advances 14, no. 33 (2024): 23840–23852, 10.1039/d4ra04784b. 39081658 PMC11287115

[63] J. Mawwa , S. U. D. Shamim , S. Khanom , M. K. Hossain , and F. Ahmed , “In‐Plane Graphene/Boron Nitride Heterostructures and Their Potential Application as Toxic Gas Sensors,” RSC Advances 11, no. 52 (2021): 32810–32823, 10.1039/d1ra06304a. 35493562 PMC9042146

[64] M. T. Ahmed , S. Islam , and F. Ahmed ,“ Density functional theory study of Mobius boron‐carbon‐nitride as potential CH_4_, H_2_S, NH_3_, COCl_2_ and CH_3_ OH gas sensor,” Royal Society Open Science 9, no. 11 (2022): 220778, 10.1098/rsos.220778. 36340512 PMC9627448

[65] M. Yang , J. Dai , L. Wang , Y. Li , and Y. Song , “First Principles Study of Structural Stability against the Distribution of Mg and Al Atoms and Adsorption Behaviors of Heavy Metals of Attapulgite,” Computational Materials Science 169 (2019): 109106, 10.1016/J.COMMATSCI.2019.109106.

[66] D. Raval , S. K. Gupta , and P. N. Gajjar , “Detection of H_2_S, HF and H_2_ Pollutant Gases on the Surface of Penta‐PdAs2 Monolayer Using DFT Approach,” Scientific Reports 13, no. 1 (2023): 1–10, 10.1038/S41598-023-27563-X. 36639684 PMC9839685

[67] D. Roy , M. R. Hossain , M. K. Hossain , M. A. Hossain , and F. Ahmed , “Density Functional Theory Study of the Sensing of Ozone Gas Molecules by Using Fullerene‐Like Group‐III Nitride Nanostructures,” Physica B: Condensed Matter 650 (2023): 414553, 10.1016/J.PHYSB.2022.414553.

[68] B. G. Fouda‐Mbanga , O. P. Onotu , and Z. Tywabi‐Ngeva , “Advantages of the Reuse of Spent Adsorbents and Potential Applications in Environmental Remediation: A Review,” Green Analytical Chemistry 11 (2024): 100156, 10.1016/j.greeac.2024.100156.

[69] S. Satyam and S. Patra , “Innovations and Challenges in Adsorption‐Based Wastewater Remediation: A Comprehensive Review,” Heliyon 10, no. 9 (2024): e29573, 10.1016/j.heliyon.2024.e29573. 38699034 PMC11064087

[70] J. Zagorac , D. Zagorac , B. Babić , T. Prikhna , and B. Matović , “Effect of Aluminum Addition on the Structure and Electronic Properties of Boron Nitride,” Journal of Solid State Chemistry 311 (2022): 123153, 10.1016/j.jssc.2022.123153.

[71] M. Noei , A. A. Salari , N. Ahmadaghaei , Z. Bagheri , and A. A. Peyghan , “DFT Study of the Dissociative Adsorption of HF on an AlN Nanotube,” Comptes Rendus Chimie 16, no. 11 (2013): 985–989, 10.1016/j.crci.2013.05.007.

[72] M. Doust Mohammadi and H. Y. Abdullah , “The Adsorption of Chlorofluoromethane on Pristine, and Al‐ and Ga‐Doped Boron Nitride Nanosheets: A DFT, NBO, and QTAIM Study,” Journal of Molecular Modeling 26, no. 10 (2020): 287, 10.1007/s00894-020-04556-5. 32980919

[73] I. Itskou , A. Kafizas , I. Nevjestic , et al., “Effects of Phosphorus Doping on Amorphous Boron Nitride's Chemical, Sorptive, Optoelectronic, and Photocatalytic Properties,” The Journal of Physical Chemistry. C, Nanomaterials and Interfaces 128, no. 31 (2024): 13249–13263, 10.1021/acs.jpcc.4c02314. 39140095 PMC11317980

[74] S. U. D. Shamim , D. Roy , S. Alam , et al., “Doubly Doped Graphene as Gas Sensing Materials for Oxygen‐Containing Gas Molecules: A First‐Principles Investigation,” Applied Surface Science 596 (2022): 153603, 10.1016/J.APSUSC.2022.153603.

[75] M. T. Ahmed , A. Al Roman , D. Roy , S. Islam , and F. Ahmed , “Phosphorus‐Doped T‐Graphene Nanocapsule toward O_3_ and SO_2_ Gas Sensing: A DFT and QTAIM Analysis,” Scientific Reports 14, no. 1 (2024): 1‐1–18, 10.1038/s41598-024-54110-z. 38342938 PMC10859388

[76] M. H. Shahria Fahim , A. Talha , S. R. Biswas , M. T. Ahmed , A. A. Roman , and D. Roy , “Adsorption Behavior of the Blue Phosphorene Monolayer, Doped with Ti and Cr, for CO_2_ and SO_3_ Gases: A First‐Principles Analysis,” Surfaces and Interfaces 72 (2025): 107318, 10.1016/j.surfin.2025.107318.

[77] Q. Tang , Z. Zhou , and Z. Chen , “Molecular Charge Transfer: A Simple and Effective Route to Engineer the Band Structures of BN Nanosheets and Nanoribbons,” Journal of Physical Chemistry C 115, no. 38 (2011): 18531–18537, 10.1021/jp2067205.

[78] R. Balu , X. Zhong , R. Pandey , and S. P. Karna , “Effect of Electric Field on the Band Structure of Graphene/Boron Nitride and Boron Nitride/Boron Nitride Bilayers,” Applied Physics Letters 100, no. 5 (2012): 174, 10.1063/1.3679174.

[79] Q. Tang , Z. Zhou , P. Shen , and Z. Chen , “Band Gap Engineering of BN Sheets by Interlayer Dihydrogen Bonding and Electric Field Control,” Chemphyschem: A European Journal of Chemical Physics and Physical Chemistry 14, no. 9 (2013): 1787–1792, 10.1002/cphc.201300141. 23606436

[80] X. Li , X. Wu , X. C. Zeng , and J. Yang , “Band‐Gap Engineering via Tailored Line Defects in Boron‐Nitride Nanoribbons, Sheets, and Nanotubes,” ACS Nano 6, no. 5 (2012): 4104–4112, 10.1021/nn300495t. 22482995

[81] W. Chen , A. Chen , R. Zhang , et al., “Strong In‐Plane Optoelectronic Anisotropy and Polarization Sensitivity in Low‐Symmetry 2D Violet Phosphorus,” Nano Letters 23 (2023): 10821. 38050812 10.1021/acs.nanolett.3c02951

[82] X. Wang , Y. Yong , W. Yang , et al., “Adsorption, Gas‐Sensing, and Optical Properties of Molecules on a Diazine Monolayer: A First‐Principles Study,” ACS Omega 6, no. 17 (2021): 11418–11426, 10.1021/acsomega.1c00432. 34056297 PMC8153939

[83] J. Y. Damte and H. Ataalite , “First‐Principles Investigation of Gas Adsorption on Bilayer Transition Metal Dichalcogenides for Sensing Toxic Gases,” Results in Physics 70 (2025): 108183, 10.1016/j.rinp.2025.108183.

[84] A. Abbasi , “DFT Study of the Electronic Properties and Gas Sensing Characteristics of the Novel Ag_2_O Modified BP/BSe Van der Waals Heterostructures,” Scientific Reports 15, no. 1 (2025): 17662, 10.1038/s41598-025-02554-2. 40399421 PMC12095626

[85] F. De Proft , C. Van Alsenoy , A. Peeters , W. Langenaeker , and P. Geerlings , “Atomic Charges, Dipole Moments, and Fukui Functions Using the Hirshfeld Partitioning of the Electron Density,” Journal of Computational Chemistry 23, no. 12 (2002): 1198–1209, 10.1002/JCC.10067. 12116389

[86] M. H. Rocky , M. Khatun , A. Al Roman , D. Roy , and M. T. Ahmed , “A DFT Study on Boron Carbon Nitride and in‐Plane Graphene‐Boron Nitride Nanosheets for O_3_ and F_2_ Gas Sensing,” Computational and Theoretical Chemistry 1237 (2024): 114639, 10.1016/J.COMPTC.2024.114639.

[87] L. Jia , J. Chen , X. Cui , Z. Wang , W. Zeng , and Q. Zhou , “Gas Sensing Mechanism and Adsorption Properties of C(2)H(4) and CO Molecules on the Ag(3)‐HfSe(2) Monolayer: A First‐Principle Study,” Frontiers in Chemistry 10 (2022): 911170, 10.3389/fchem.2022.911170. 35646821 PMC9133379

[88] A. Talha , F. H. Shihab , M. T. Ahmed , A. Al Roman , Z. Kowser , and D. Roy , “Density Functional Theory Study of the Adsorption and Dissociation of OF_2_ and O_3_ Gases on the Surface of Pristine and Al, Ti and Cr Doped Graphene,” AIP Advances 14, no. 7 (2024): 75008, 10.1063/5.0214735.

[89] Y. S. Al‐Hamdani , D. Alfé , O. A. von Lilienfeld , and A. Michaelides , “Tuning Dissociation Using Isoelectronically Doped Graphene and Hexagonal Boron Nitride: Water and Other Small Molecules,” The Journal of Chemical Physics 144, no. 15 (2016): 154706. 27389233 10.1063/1.4945783

[90] K. Seki and S. Yunoki , “Brillouin‐Zone Integration Scheme for Many‐Body Density of States: Tetrahedron Method Combined with Cluster Perturbation Theory,” Physical Review B 93, no. 24 (2016): 245115, 10.1103/PHYSREVB.93.245115.

[91] H. Li , H. Yu , X. Quan , S. Chen , and Y. Zhang , “Uncovering the Key Role of the Fermi Level of the Electron Mediator in a Z‐Scheme Photocatalyst by Detecting the Charge Transfer Process of WO_3_‐Metal‐gC_3_N_4_ (Metal = Cu, Ag, Au,” ACS Applied Materials and Interfaces 8, no. 3 (2016): 2111–2119, 10.1021/acsami.5b10613. 26728189

[92] R. D. Daymond , F. H. Shihab , N. Siddique , M. T. Ahmed , A. Al Roman , and D. Roy , “Al and Ti‐Doped Black Phosphorus as Sensitive Materials for Adsorption of HF and H_2_S Toxic Gases: An Ab Initio Study,” RSC Advances 15, no. 35 (2025): 28703–28720, 10.1039/d5ra04844c. 40861972 PMC12376769

[93] J. H. Li , J. Wu , and Y. X. Yu , “DFT Exploration of Sensor Performances of Two‐Dimensional WO_3_ to Ten Small Gases in Terms of Work Function and Band Gap Changes and I‐V Responses,” Applied Surface Science 546 (2021): 149104, 10.1016/J.APSUSC.2021.149104.

[94] M. G. Muktadir , A. Alam , A. A. Piya , and S. U. D. Shamim , “Exploring the adsorption ability with sensitivity and reactivity of C_12_ –B_6_N_6_, C_12_ –Al_6_N_6_, and B_6_N_6_ –Al_6_N_6_ heteronanocages towards the cisplatin drug: a DFT, AIM, and COSMO analysis,” RSC Advances 12, no. 45 (2022): 29569, 10.1039/D2RA04011E. 36320781 PMC9578514

[95] K. Hossain , M. T. Ahmed , R. A. Rabu , and F. Ahmed , “First‐Principles Investigations of As‐Doped Tetragonal Boron Nitride Nanosheets for Toxic Gas Sensing Applications,” Nanoscale Advances 7, no. 1 (2024): 354–369, 10.1039/D4NA00739E. 39629350 PMC11610605

[96] X. Peng , D. Liu , F. Zhao , and C. Tang , “Gas Sensing Properties of Mg‐Doped Graphene for H_2_S, SO_2_, SOF_2_, and SO_2_F_2_ Based on DFT,” International Journal of Quantum Chemistry 122, no. 22 (2022): e26989, 10.1002/QUA.26989;JOURNAL: JOURNAL: 1097461X;WGROUP: STRING: PUBLICATION.

[97] M. Rezvani , M. Astaraki , A. Rahmanzadeh , and M. Darvish Ganji , “Theoretical Assessments on the Interaction between Amino Acids and the g‐Mg_3_N_2_ Monolayer: Dispersion Corrected DFT and DFT‐MD Simulations,” Physical Chemistry Chemical Physics 23, no. 32 (2021): 17440–17452, 10.1039/D1CP02891J. 34352060

[98] F. H. Shihab , M. F. Mou , D. Roy , A. Al Roman , and M. T. Ahmed , “B_4_C_4_ Nanocluster ring for Toxic Heavy Metal Ion Adsorption from Wastewater: A DFT Study,” Journal of Physics and Chemistry of Solids 207, no. May (2025): 112896, 10.1016/j.jpcs.2025.112896.

[99] S. Tan , R. Li , H. Yuan , L. Chen , J. Zeng , and T. Jiang , “Adsorption and Gas Sensing Properties of Pdn(n=1–3) Cluster‐Modified PtSe_2_ on Transformer Fault Characterizing Gases Under Biaxial Strain and Electric Field,“ Colloids and Surfaces A: Physicochemical and Engineering Aspects 697 (2024): 134500, 10.1016/J.COLSURFA.2024.134500.

[100] R. M. Jamila , S. Narasimman , A. Prasanth , M. Muthukumar , Z. C. Alex , and G. T. Anand , “Fiber Optic Sensor Coated with Multiple Layers of Hexagonal Boron Nitride Nanosheets (BNNS) for the Detection of Volatile Organic Compounds,” ACS Applied Materials \& Interfaces 16 (2024): 35525. 38934269 10.1021/acsami.4c05230

[101] T. Saiki , S. Ohkubo , K. Funahashi , T. Yamazaki , T. Kajita , and T. Katsufuji , “Change in the Optical Spectrum of BaV1_0_O_15_ with Applied Uniaxial Strain,” Physical Review. B 101, no. 12 (2020): 121111, 10.1103/PhysRevB.101.121111.

[102] Y‐X. Li , S‐S. Chen , and F‐D. Ren , “Theoretical Insights into the Structures and Mechanical Properties of HMX/NQ Cocrystal Explosives and Their Complexes, and the Influence of Molecular Ratios on Their Bonding Energies,” Journal of Molecular Modeling 21, no. 9 (2015): 1–12, 10.1007/S00894-015-2790-2/METRICS. 26318201

[103] X. D. D. Dexlin , J. D. D. Tarika , S. M. Kumar , A. Mariappan , and T. J. Beaula , “Synthesis and DFT Computations on Structural, Electronic and Vibrational Spectra, RDG Analysis and Molecular Docking of Novel Anti COVID‐19 Molecule 3, 5 Dimethyl Pyrazolium 3, 5 Dichloro Salicylate,” Journal of Molecular Structure 1246, no. 5 (2021): 131165, 10.1016/J.MOLSTRUC.2021.131165. 36532120 PMC9749904

[104] J. Quan , B. Teng , X. Wen , Y. Zhao , R. Liu , and M. Luo , “Hydrogen Fluoride Adsorption and Reaction on the α‐Al2O30001 Surface: A Density Functional Theory Study,” The Journal of Chemical Physics 136 (2012): 114701, 10.1063/1.3694102. 22443784

[105] S. N. Ema , M. Khaleque , A. Ghosh , A. A. Piya , U. Habiba , and S. U. D. Shamim , “Surface Adsorption of Nitrosourea on Pristine and Doped (Al, Ga and In) Boron Nitride Nanosheets as Anticancer Drug Carriers: The DFT and COSMO Insights,” RSC Advances 11 (2021): 36866–36883, 10.1039/d1ra07555a. 35494400 PMC9043538

[106] M. Esrafili , P. Mousavian , and F. A. Rad , “Adsorption of Formamide over Pristine and Al‐Doped Boron Nitride Nanosheets: A Dispersion‐Corrected DFT Study,” Journal of Molecular Graphics & Modelling 82 (2018): 101–107, 10.1016/j.jmgm.2018.04.004. 29723820

[107] L. Chhana , B. Lalroliana , R. C. Tiwari , et al., “Theoretical Study of ZnS Monolayer Adsorption Behavior for CO and HF Gas Molecules,” ACS Omega 7, no. 44 (2022): 40176–40183, 10.1021/acsomega.2c05064. 36385877 PMC9648164

[108] S. Pervaiz , M. U. Saeed , S. Khan , et al., “Highly Sensitive Sensing of CO and HF Gases by Monolayer CuCl,” RSC Advances 14, no. 23 (2024): 16284–16292, 10.1039/D4RA01519C. 38774614 PMC11106810

[109] B. A. Kalwar , W. Fangzong , A. M. Soomro , M. R. Naich , M. H. Saeed , and I. Ahmed , “Highly sensitive work function type room temperature gas sensor based on Ti doped hBN monolayer for sensing CO_2_, CO, H_2_ S, HF and NO. A DFT study,” RSC Advances 12, no. 53 (2022): 34185–34199, 10.1039/d2ra06307g. 36545633 PMC9709776

[110] A. Zhang , Q. Dong , Y. Gui , J. Li , and F. Wan , “Gas‐Sensing Property of TM‐MoTe_2_ Monolayer towards SO_2_, SOF_2_, and HF Gases,” Molecules 27, no. 10 (2022): 3176, 10.3390/molecules27103176. 35630656 PMC9147850

[111] D. Raval , S. K. Gupta , and P. N. Gajjar , “Detection of H_2_S, HF and H_2_ Pollutant Gases on the Surface of Penta‐PdAs_2_ Monolayer Using DFT Approach,” Scientific Reports 13, no. 1 (2023): 699, 10.1038/s41598-023-27563-x. 36639684 PMC9839685

